# Modulating Lysine Crotonylation in Ulcerative Colitis Maintains Mitochondrial Homeostasis: Modulating Crotonylation in Ulcerative Colitis

**DOI:** 10.1002/EXP.20240129

**Published:** 2025-09-19

**Authors:** Tongtong Liu, Binyan Lin, Ying Zhang, Jiayu Su, Xiaochao Hu, Xuan Wang, E‐Hu Liu, Shijia Liu

**Affiliations:** ^1^ Department of Pharmacy Affiliated Hospital of Nanjing University of Chinese Medicine Jiangsu Province Hospital of Chinese Medicine Nanjing China; ^2^ College of The First Clinical Medicine Nanjing University of Chinese Medicine Nanjing China; ^3^ Laboratory of Herbal Active Compound Omics and Target Identification, School of Pharmacy Nanjing University of Chinese Medicine Nanjing China

**Keywords:** Citrate synthetase, Lysine crotonylation, Mitophagy, Ulcerative colitis

## Abstract

Ulcerative colitis (UC) is a chronic and persistent clinical condition that is challenging to cure. Lysine crotonylation (KCr), a recently discovered post‐translational modification (PTM), alters protein structure, stability, localization and activity in a variety of processes including cell differentiation and organism development. This study was designed to elucidate the pathophysiological relevance of KCr in UC and uncover potential underlying mechanisms involved. PTM proteomics was employed to track dynamic alterations in KCr sites and protein level in the colon tissue of dextran sulfate sodium (DSS)‐induced UC model mice. Following the validation of differentially crotonylated proteins via Western blot assay, functional and mechanistic analyses of specific KCr sites were conducted in vitro. Gain‐of‐function or loss‐of‐function mutations were implemented at selected protein KCr sites. The differentially crotonylated proteins including citrate synthetase (CS) between the colon tissue of DSS‐induced mice and control mice were predominantly associated with the tricarboxylic acid (TCA) cycle, as evidenced by significant enrichment in the KEGG pathway analysis. These proteins were primarily localized in mitochondria, suggesting a potential link among UC pathogenesis, mitochondria and the TCA cycle. Collectively, increased KCr restricts inflammasome activation by inducing mitophagy, thereby maintaining mitochondrial homeostasis, reducing oxidative stress and inhibiting apoptosis in UC. KCr represents a potential promising therapeutic target for the treatment of UC.

## Graphical abstract

NaCr promotes KCr to impede the inflammatory progression of ulcerative colitis. KCr induces mitophagy by activating the PINK1/PARKIN pathway to restrict NLRP3 inflammasome activation, thereby decreasing the production and release of various inflammatory cytokines, such as IL‐1β and TNF‐α. KCr induces mitophagy and inhibits NLRP3 inflammasome activation via the continuous activation of K375 site in citrate synthetase.

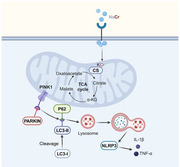

## 1 Introduction

Ulcerative colitis (UC) is an inflammatory bowel disease (IBD) characterized by diffusive intestinal inflammation of the mucosal membranes [1]. In recent years, the incidence of UC has increased globally, especially in developing countries [2]. The typical symptoms of UC include hematochezia, abdominal pain and tenesmus. Patients with UC have an increased risk of developing colitis‐associated colon cancer [3]. At present, the underlying pathogenesis of UC is still incompletely understood and is mainly related to immune disorders, intestinal flora dysbiosis, epithelial barrier defects, genetics and the environment [4, 5]. The medical management of UC involves 5‐amino‐salicylate, corticosteroids, thiopurine, immunosuppressants, fecal microflora transplantation and surgery [6]. Although current medications have provided some therapeutic benefits, certain patients with UC may still experience adverse effects such as nausea, indigestion, headache, vomiting, and abdominal pain [7]. Hence, there is a pressing need for novel pathophysiology‐targeted therapies.

Mitochondria are key for bioenergy and biosynthesis, crucial for cell, and human health [8]. Maintaining mitochondrial homeostasis within the gastrointestinal epithelium is key to ameliorating UC [9]. Optimal tricarboxylic acid (TCA) cycle function maintains this homeostasis, balancing redox and preventing oxidative stress disruptions [10]. Imbalances in enzyme activity within the TCA cycle disrupt mitochondrial homeostasis, leading to energy deficiencies, heightened oxidative stress and metabolic irregularities linked to various diseases [11]. Citrate synthase (CS), a TCA cycle rate‐limiting enzyme, is essential for TCA cycle function and mitochondrial homeostasis [12]. Impaired enzyme (such as CS) function disrupts the TCA cycle and reduces energy production. Consequently, cells initiate mitophagy to eliminate dysfunctional mitochondria and sustain regular physiological processes. Mitophagy is a type of selective autophagy that is responsible for degrading dysfunctional or excess mitochondria to maintain mitochondrial homeostasis. Mitophagy plays a pivotal role in numerous diseases, particularly UC [13]. In patients with UC, mitochondrial dysfunction, elevated oxidative stress and persistent inflammatory reaction will trigger the activation of mitophagy. In UC, mitochondrial damage induces the accumulation of PTEN induced kinase 1 (PINK1) at the outer mitochondrial membrane, stimulates the activation and recruitment of Parkin RBR E3 ubiquitin protein ligase (PARKIN) to initiate mitophagy. Subsequent ubiquitination leads to the accumulation of receptor proteins such as ubiquitin‐binding protein (P62) on the outer mitochondrial membrane, facilitates the recruitment of ubiquitinated material to autophagosomes through binding to microtubule‐associated protein 1 light chain 3 (LC3). The fusion of mature autophagosomes with lysosomes results in the formation of autophagolysosomes, ultimately leading to the degradation of engulfed mitochondria. A recent study highlighted the close relationship between mitophagy and UC [14]. The interplay among mitochondrial TCA cycle enzymes, mitochondrial homeostasis and mitophagy in UC remains ambiguous.

Post‐translational modification (PTM) is a major regulatory mechanism that precisely controls protein function in a variety of biological processes [15], including cell differentiation and organism development, by conferring new properties to modified proteins [16]. Abnormal protein modifications lead to diseases such as UC, Crohn's disease and colorectal cancer [17]. Among the 20 ribosomally encoded amino acid residues, lysine is the most commonly subjected to PTM, leading to significant functional and regulatory effects [18]. Lysine crotonylation (KCr) is a newly discovered protein PTM that affects protein structure, stability, localization and activity [19]. KCr plays important roles in a variety of disease processes, such as acute kidney injury, depression, HIV latency and cancer [20, 21]. Histone KCr was first identified as a new type of PTM in 2011 [22], serving as a critical signal that regulates the differentiation of male germ cells. Subsequent research indicated that crotonylation of non‐histone K residues had effect on metabolism, cell cycle progression and cellular organization [23]. Despite these discoveries, the role of KCr in the pathological mechanisms of UC remains poorly understood.

In this study, a dextran sulfate sodium (DSS)‐induced UC mouse model was established, the alterations of crotonylation were identified through PTM proteomics. Sodium crotonate (NaCr) was chosen as a KCr donor for the experiments. NaCr‐induced KCr enhancement mitigated DSS‐induced weight loss, colon length reduction and colonic damage. Additionally, enhanced KCr notably reduced the serum level of proinflammatory cytokines. Elevated KCr level significantly improved mitochondrial structural damage and reduced DSS‐induced cell apoptosis. Increased KCr level also led to a reduction in malondialdehyde (MDA) level, an elevation in superoxide dismutase (SOD) activity and an increase in glutathione (GSH) level, suggesting that the increase in KCr alleviated UC by preserving mitochondrial homeostasis, reducing oxidative stress and inhibiting apoptosis. Moreover, KCr‐induced mitophagy contributed to the activation of the PINK1/PARKIN signaling pathway and suppressed activation of NLRP3 inflammasome both in vivo and in vitro. Notably, KCr‐mediated inhibition of the NLRP3 inflammasome was reversed by the mitophagy inhibitor chloroquine (CQ) and mitochondrial division inhibitor 1 (Mdivi‐1), indicating that KCr limited NLRP3 inflammasome activation by promoting mitophagy. Ultimately, KCr induced mitophagy and inhibited NLRP3 inflammasome activation through the K375 site in CS. Overall, our findings highlighted the role of KCr in suppressing NLRP3 inflammasome activation and suggested that increasing KCr may be a potential therapeutic approach for the treatment of UC.

## 2 Materials and Methods

### 2.1 Chemicals and Reagents

DSS (MP Biomedicals, Lot: S2839); anti‐crotonyllysine antibody (Jingjie PTM BioLab, PTM‐502; Lot: 1037267K306; 1:1000 dilution); anti‐succinyllysine antibody (PTM‐419; Lot: 1D05032317L804; 1:1000 dilution); anti‐malonyllysine antibody (PTM‐902; Lot: 23056103K312; 1: 1000 dilution); anti‐β‐hydroxybutyryllysine antibody (PTM‐1201RM; Lot: RL053142; 1:200 dilution); anti‐lactyllysine antibody (PTM‐1401RM; Lot: RL063009; 1:300 dilution); NaCr (Shanghai yuanye Bio‐Technology, Cas No.14379‐00‐1); LEGENDplex multi‐analyte flow assay kit (BioLegend, Cat No.741043); TUBULIN antibody (Proteintech, Cat No. 11224‐1‐AP; 1:5000 dilution); GAPDH antibody (Proteintech, Cat No. 60004‐1‐Ig; 1:5000 dilution); BAX antibody (Proteintech, Cat No. 60267‐1‐Ig; 1:5000 dilution); BCL2 antibody (Proteintech, Cat No. 68103‐1‐Ig; 1:2000 dilution); CS antibody (Abcam, ab129095; 1:1000 dilution); PINK1 antibody (Abcam, ab216144; 1:1000 dilution); PARKIN antibody (Abcam, ab77924; 1:2000 dilution); LC3B antibody (Abcam, ab192890; 1:2000 dilution); P62 antibody (Abcam, ab109012; 1:10000 dilution); NLRP3 antibody (Abcam, ab263899; 1:1000 dilution); primer synthesis (Beijing Tsingke Biotech Co., Ltd).

### 2.2 Animal Experiments and Ethics Statement

All animal experiments were authorized by the Ethics Committee of Nanjing University of Chinese Medicine Affiliated Hospital (Approval No. 2023NL‐KS222). Female and male C57BL/6 mice were procured from Jiangsu Huachuangxinnuo Pharmaceutical Technology Co., Ltd. (Certificate No. 202332458), and underwent a 7‐day acclimatization period. Except for those in the control group, mice aged 6–8 weeks were provided with 3% DSS in their drinking water for 7 days and then sacrificed on the seventh day. In the pre‐experiments, we employed NaCr at a dosage of 10 or 20 mg kg^−1^, with the group treated at a dosage of 20 mg kg^−1^ demonstrating a more potent antagonistic effect on UC. Mice were gavaged with NaCr or CMC‐Na (0.5%). Body weight, fecal occult blood, and DAI were monitored daily. 24 h after the final treatment, the mice were humanely euthanized by cervical dislocation. Colon tissue and serum samples were collected for following analysis. This study adhered to ethical guidelines and rigorous scientific protocols and met the standards set by the Ethics Committee for Animal Experimentation.

### 2.3 Procedures for the PTM Proteomic Analysis

Proteomic analysis was conducted by Jingjie PTM BioLab, Co., Ltd., in China. Quantitative crotonylation modification omics analysis involved a combination of TMT labeling, HPLC fractionation, crotonylation and mass spectrometry‐based quantitative proteomics. WoLF PSORT software was utilized for predicting and categorizing the sublocalization of differentially modified proteins.

### 2.4 Cytokine Analysis

Serum samples were utilized to analyze the level of IL‐6, TNF‐α, IL‐10, IL‐1β and other inflammatory factors following the instructions provided with the LEGENDplex multifactor kit (USA). Analysis was performed using Cytoflex flow cytometry.

### 2.5 Cell Culture, Treatment and Plasmid Transfection

Human normal intestinal epithelial NCM460 cell line was cultured in DMEM with 1% penicillin‐streptomycin, 10% fetal bovine serum (ABW, China) in a 5% CO_2_ humidified atmosphere at 37°C. Lipopolysaccharide (LPS, 2 µg mL^−1^, Sigma Aldrich, USA) was utilized to induce inflammation in NCM460 cells. CQ (10 µm, TargetMol, China) and Mdivi‐1 (10 µm, OriLeaf, China) were used to inhibit mitophagy. The human CS coding sequences, along with the CS K375R (substitution of AAG with AGA) and CS K375Q (substitution of AAG with CAG) mutants, were synthesized, cloned into the pGCMV/MCS/EGFP/Neo vector, and transfected into cells using Lipo8000 for 24 h under standard conditions.

### 2.6 Quantitative Polymerase Chain Reaction (qPCR)

Total RNA was isolated from cells or colon tissues using a cell/tissue total RNA kit (Vazyme Biotech Co., Ltd, China). Subsequently, the RNA samples were quantified and assessed for purity using a DS‐11FX spectrophotometer (Denovix, USA). Reverse transcription was performed using a first‐strand cDNA synthesis kit (Vazyme Biotech Co., Ltd, China). qPCR analysis was carried out on a QuantStudio3 PCR detection system (Thermo Fisher Scientific, USA), and the results were analyzed using the 2^−ΔΔ^ *^CT^* method. The primer sequences for qPCR (Table 1) were synthesized by Tsingke Biotechnology Co., Ltd., (China).

**TABLE 1:** qPCR primer sequences (F stands for forward primers, R stands for reverse primers).

Gene	Source	Primer sequence (5′–3′)
*Pink1*	Mouse	F: GGCTTCCGTCTGGAGGATTAT R: AACCTGCCGAGATATTCCACA
*Parkin*	Mouse	F: CCAGAGGAAAGTCACCTGCGAA R: GTTCGAGCAGTGAGTCGCAATC
*Lc3*	Mouse	F: TTATAGAGCGATACAAGGGGGAG R: CGCCGTCTGATTATCTTGATGAG
*P62*	Mouse	F: ATGTGGAACATGGAGGGAAGA R: GGAGTTCACCTGTAGATGGGT
*Nlrp3*	Mouse	F: TCACAACTCGCCCAAGGAGGAA R: AAGAGACCACGGCAGAAGCTAG
*Cs*	Mouse	F: GGACAATTTTCCAACCAATCTGC R: TCGGTTCATTCCCTCTGCATA
*β‐actin*	Mouse	F: GGCTGTATTCCCCTCCATCG R: CCAGTTGGTAACAATGCCATGT
*IL‐6*	Human	F: ACTCACCTCTTCAGAACGAATTG R: CCATCTTTGGAAGGTTCAGGTTG
*IL‐1β*	Human	F: AGCTACGAATCTCCGACCAC R: CGTTATCCCATGTGTCGAAGAA
*TNF‐α*	Human	F: CCTCTCTCTAATCAGCCCTCTG R: GAGGACCTGGGAGTAGATGAG
*PINK1*	Human	F: CCCAAGCAACTAGCCCCTC R: GGCAGCACATCAGGGTAGTC
*PARKIN*	Human	F:CCAGAGGAAAGTCACCTGCGAA R:CTGAGGCTTCAAATACGGCACTG
*LC3*	Human	F: AAGGCGCTTACAGCTCAATG R: CTGGGAGGCATAGACCATGT
*P62*	Human	F: GCACCCCAATGTGATCTGC R: CGCTACACAAGTCGTAGTCTGG
*NLRP3*	Human	F: GGACTGAAGCACCTGTTGTGCA R: TCCTGAGTCTCCCAAGGCATTC
*CS*	Human	F: TGCTTCCTCCACGAATTTGAAA R: CCACCATACATCATGTCCACAG
*GAPDH*	Human	F: GGAGCGAGATCCCTCCAAAAT R: GGCTGTTGTCATACTTCTCATGG

### 2.7 HPIC‐MRM‐MS Analysis

The HPIC separation was performed using a Thermo Scientific Dionex ICS‐6000 HPIC System, utilizing Dionex Ion Pac AS11‐HC (2 × 250 mm) and AG11‐HC (2 mm × 50 mm) columns. Mobile phase A consisted of 100 mm NaOH in water, while mobile phase B was ultrapure water. An additional pumping system delivered a solvent mixture (2 mm acetic acid in methanol) before entering the electrospray ionization (ESI) source at a flow rate of 0.15 mL min^−1^. The column temperature was maintained at 30°C. The autosampler temperature was set to 4°C, with an injection volume of 5 µL.

For assay development, an AB SCIEX 6500 QTRAP^+^ triple quadrupole mass spectrometer with an ESI interface was employed. The typical ion source parameters included an ion spray voltage of −4500 V, temperature of 450°C, ion source gas 1 at 45 psi, ion source gas 2 at 45 psi, and curtain gas at 30 psi. The multiple reaction monitoring (MRM) parameters for each targeted analyte were optimized via flow injection analysis.

### 2.8 Observation of Mitochondrial Morphology

Transmission electron microscopy (TEM) was utilized to examine the ultrastructure of mitochondria in the colon tissue of DSS‐induced mice.

### 2.9 Determination of Oxidative Stress Index

The level of MDA, SOD, and GSH in the serum of DSS‐induced mice was quantified by a colorimetric assay following the provided instructions.

### 2.10 Western Blot

Proteins from colon tissue or cells were extracted using a total protein extraction kit (Yeasen Biotechnology Co., Ltd., China), and the protein concentration was determined using the BCA method (Yeasen Biotechnology Co., Ltd., China). Subsequently, the proteins were separated by 10% SDS‐PAGE and transferred to a nitrocellulose membrane using a Bio‐Rad Trans‐Blot system. Following staining with Lichunhong dye, the bands were washed thrice with PBST. The membranes were then blocked with 5% skimmed milk and incubated overnight at 4°C with primary antibodies (TUBULIN, GAPDH, BAX, BCL2, BAD, BAK, PINK1, PARKIN, LC3, P62, NLRP3, CS). Afterward, the membranes were probed with the secondary antibody for 60 min. Finally, the target proteins were visualized using ECL reagent and quantified with ImageJ software (NIH Image, USA).

### 2.11 Statistical Analysis

Data collected from ≥3 independent assays were presented as mean ± standard deviation (SD). Results were analyzed using a Prism 9.0 software (GraphPad, USA). Comparison between two groups were conducted using the unpaired *t* test (two‐tailed). Comparison among multiple groups were conducted using one‐way ANOVA and two‐way ANOVA. *P* < 0.05 was considered statistically significant.

## 3 Results

### 3.1 Lower KCr Level of Enzymes in the TCA Cycle is Associated With UC

**FIGURE 1:**
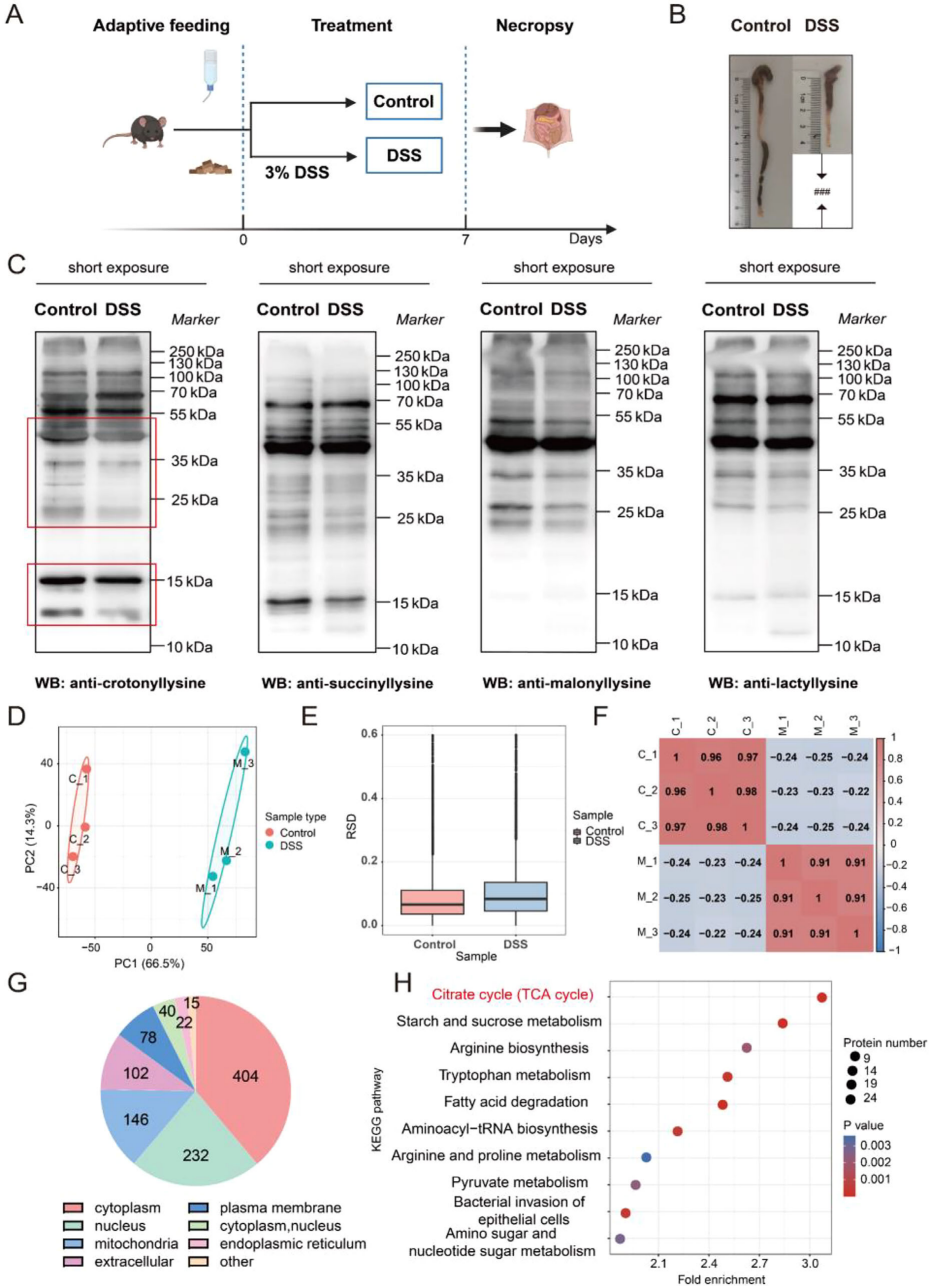
Lower KCr level of enzymes in the TCA cycle is associated with UC. (A) Schematic overview of the DSS‐induced UC model. (B) Representative pictures of mice colon. (C) Representative Western blot images of PTMs in the colon tissue between control and DSS‐induced UC mice. (D) Two‐dimensional scatter plot of PCA distribution. (E) Box plot of RSD distribution. (F) Heatmap of PCC from all quantified proteins. (G) Differential protein sublocalization. (H) KEGG pathway enrichment. ^###^ *p* < 0.001 versus control group (*n* = 6).

To establish an acute UC mouse model, mice were treated with 3% DSS (wt/vol) for 7 days [24] (Figure 1A). Compared with those of the control group, the colons of the DSS‐induced UC group were markedly shorter and more atrophic (Figure 1B). To determine the differences in PTMs between control and DSS‐induced UC mice, we assessed the level of crotonylation, succinylation, malonylation, and lactylation in the colon tissues of the mice. The analysis revealed a significant alteration in the crotonylation level in colon tissue between the DSS‐induced UC group and control group (Figure 1C and Figure S1). Next, we used proteomics to analyze the differences in the crotonylation level between the UC group and control group. Principal component analysis (PCA), relative standard deviation (RSD) and Pearson's correlation coefficient (PCC) were used to evaluate the quantitative repeatability of the modifications (Figure 1D–F). As shown in Figure 1G, the top three sublocalizations of the differentially modified proteins were in the cytoplasm (404), nucleus (232) and mitochondria (146). The differentially modified proteins were selected for KEGG enrichment analysis, and there was statistically significant enrichment of the differentially modified proteins in the TCA cycle pathway (Figure 1H). Through proteome‐wide enrichment analysis, proteins modified by KCr, such as 2‐hydroxyglutarate dehydrogenase (DLST), CS and succinyl‐CoA ligase, were found to be sublocalized in mitochondria. Alterations of KCr in UC may be linked to changes in mitochondrial enzymes and mitochondrial homeostasis. The level of these proteins was lower in DSS‐induced UC mice than in control mice, and all of them were modified by KCr and sublocalized primarily in mitochondria (Table 2). Therefore, it is postulated that lower KCr level of enzymes in the TCA cycle is associated with UC.

**TABLE 2:** Statistics of KCr differentially modified proteins in the TCA cycle. (*p* < 0.05, FC < 1/1.5).

Gene Name	Description	Modification site	DSS versus control	Sublocalization
*DLST*	2‐hydroxyglutarate dehydrogenase	K218	0.325	Mitochondria
*CS*	Citrate synthetase	K375	0.352	Mitochondria
*SUCLG1*	Succinyl‐CoA ligase	K66	0.427	Mitochondria
*OGDH*	2‐oxoglutarate dehydrogenase	K564	0.441	Mitochondria
*MDH1*	Malate dehydrogenase	K122	0.481	Cytoplasm
*MDH1*	Malate dehydrogenase	K201	0.494	Cytoplasm
*CS*	Citrate synthetase	K76	0.519	Mitochondria
*CS*	Citrate synthetase	K366	0.536	Mitochondria
*ACO2*	Aconite hydratase	K573	0.551	Mitochondria
*IDH3A*	Isocitrate dehydrogenase	K326	0.566	Cytoplasm
*MDH2*	Malate dehydrogenase	K78	0.582	Mitochondria
*ACO2*	Aconite hydratase	K517	0.587	Mitochondria
*OGDH*	2‐oxoglutarate dehydrogenase	K860	0.599	Mitochondria
*IDH2*	Isocitrate dehydrogenase	K263	0.601	Mitochondria
*SDHA*	Succinate dehydrogenase	K485	0.602	Mitochondria
*FH*	Fumarate hydratase	K112	0.613	Mitochondria
*PC*	Pyruvate carboxylase	K1109	0.627	Mitochondria

### 3.2 NaCr Promotes KCr to Impede the Inflammatory Progression of UC

**FIGURE 2:**
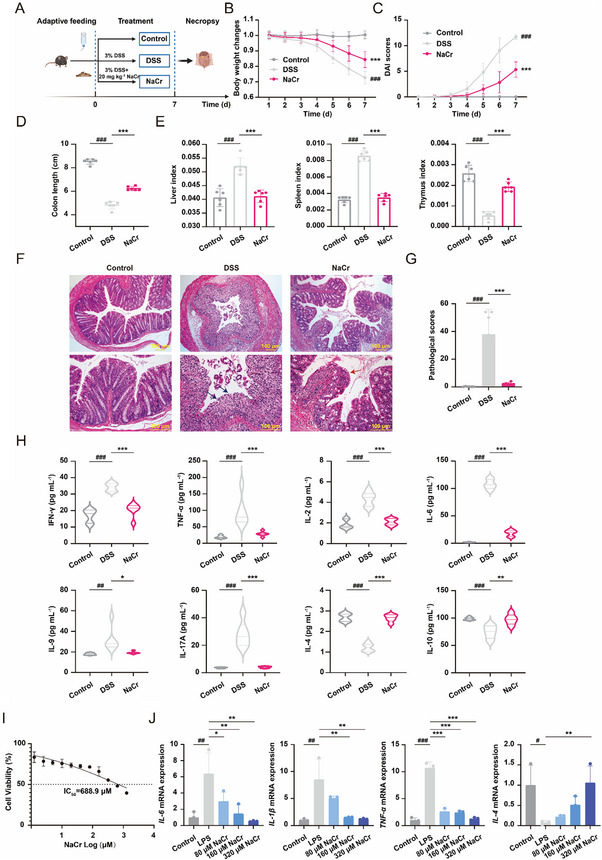
NaCr promotes KCr to impede the inflammatory progression of UC. (A) Timeline of the animal experiment. Female mice in NaCr group were treated with NaCr (20 mg kg^−1^). (B) Body weight changes (*n* = 12). (C) DAI scores (*n* = 12). (D) Length of colon tissues (*n* = 6). (E) Liver index, spleen index and thymus index (*n* = 6). (F) H&E‐staining. (G) Pathological scores (*n* = 6). The blue arrows represent the complete destruction of ulcers and crypt structure. The red arrow indicates mucosal repair and reduced crypt damage. (H) The contents of IFN‐γ, TNF‐α, IL‐2, IL‐6, IL‐9, IL‐17A, IL‐4, and IL‐10 in serum were detected by ELISA assay (*n* = 6). (I) NCM460 cells viability was detected using CCK8 assay at 48 h after NaCr treatment. (J) NCM460 cells were treated with LPS (2 µg mL^−1^). The mRNA expression of proinflammatory cytokines (IL‐6, IL‐1β, TNF‐α, and IL‐4) was determined by qPCR (*n* = 3). Data were presented as the mean ± SD. *p < 0.05, **p < 0.01, ***p < 0.001 versus DSS group; ^##^ *p* < 0.01, ^###^ *p* < 0.001 versus control group.

Given the reduced enrichment of KCr observed in DSS‐induced UC mice, we hypothesized that enhancing the general KCr level would be beneficial for alleviating UC. NaCr, a donor for KCr, has been shown to increase the level of KCr both in vivo and in vitro [25]. Also, we confirmed that NaCr promoted KCr as a donor (Figure S2). To evaluate the role of NaCr in the development of UC, we generated DSS‐induced UC mice and gavaged them with CMC‐Na (solvent control), DSS, and NaCr (DSS + NaCr) (Figure 2A). The colon, liver, spleen, thymus tissues, and serum of the mice were collected for subsequent experiments after 7 days treatment. As shown in Figure 2B–D, NaCr‐induced KCr enhancement alleviated the body weight loss, DAI scores increase and reduction in colon length induced by DSS described above. Also, KCr enhancement had a rehabilitative impact on liver, spleen and thymus indices (Figure 2E). KCr restored DSS‐induced damage to the morphology of colon villi and crypts (Figure 2F). Notably, NaCr robustly reduced the pathological score in DSS‐induced UC mice, as determined by H&E staining (Figure 2G). These results indicate that KCr alleviates the histopathological injury in UC mice. Furthermore, KCr can impede the inflammatory progression of UC when NaCr is administered after the establishment of DSS‐induced UC model without any discernible gender‐based differences or associated toxicity, and administration of NaCr at 20 mg kg^−1^ exerted a superior anti‐inflammatory effect in UC (Figure S3–S6).

The inflammatory response in UC is orchestrated by a multitude of cytokines, including proinflammatory cytokines (such as IFN‐γ, TNF‐α, IL‐1β, IL‐2, IL‐6, IL‐9, and IL‐17A) and anti‐inflammatory cytokines (such as IL‐4 and IL‐10) [26]. Compared with those in the control group, the contents of IFN‐γ, TNF‐α, IL‐2, IL‐6, IL‐9, and IL‐17A in the DSS‐induced UC group were significantly elevated, while the contents of the anti‐inflammatory factors IL‐4 and IL‐10 were significantly reduced. Treatment with NaCr reversed the inflammatory cytokine level changes induced by DSS (Figure 2H). In addition, NCM460 cells were treated with LPS (2 µg mL^−1^) for 24 h to establish an in vitro inflammation model [27]. We selected 80, 160, and 320 µm as treatment concentrations of NaCr according to the results of the CCK8 assay (Figure 2I). We found that NaCr reversed the upregulation of IL‐6, IL‐1β, TNF‐α, and the downregulation of IL‐4 mRNA expression induced by LPS in NCM460 cells in a concentrations‐dependent manner (Figure 2J). The above results suggest that KCr enhancement impedes the development of UC both in vivo and in vitro.

### 3.3 Increased KCr in CS Enhances the Production of Citrate Catalyzed by CS

**FIGURE 3:**
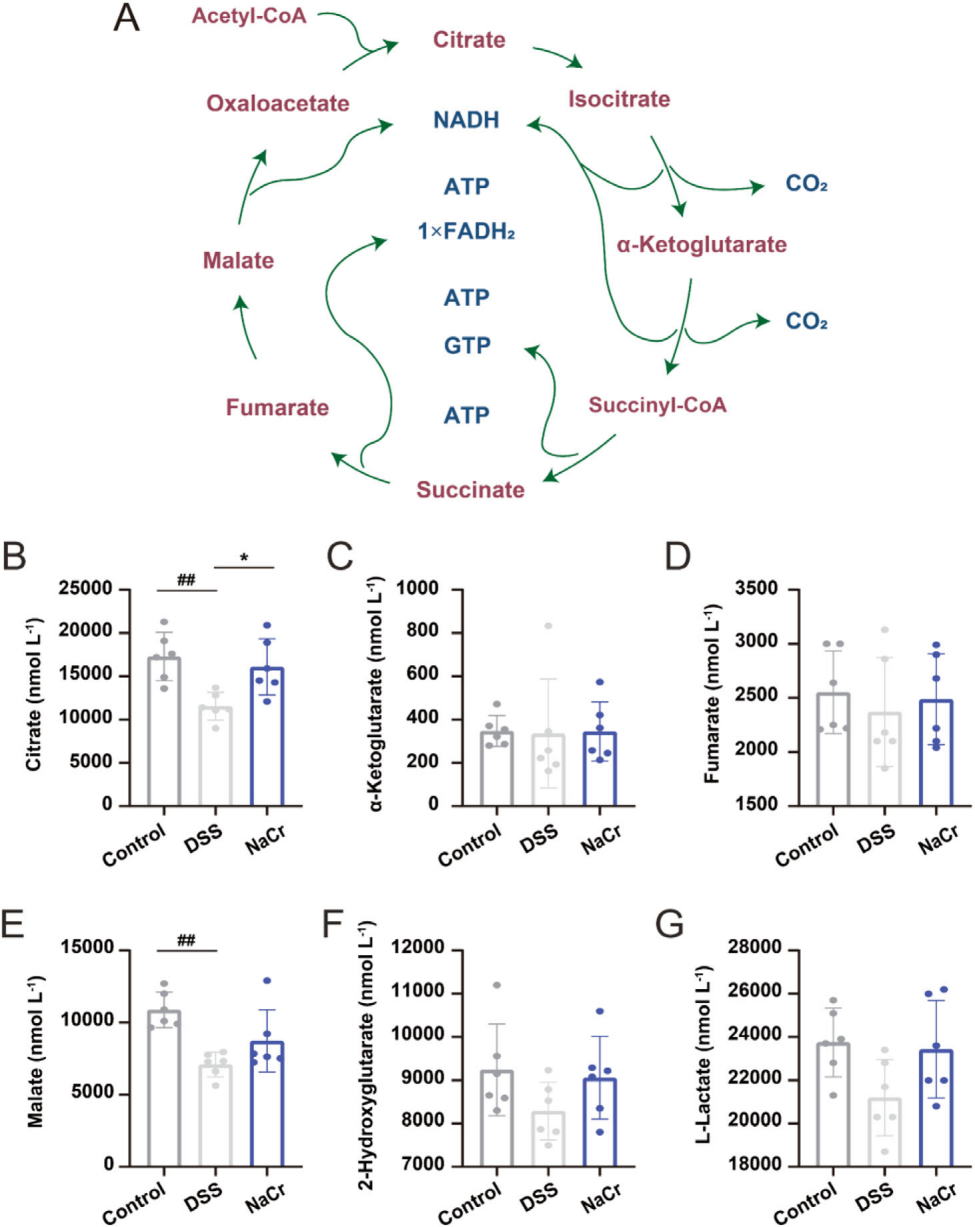
Increased KCr in CS enhances the production of citrate catalyzed by CS. (A) Metabolites in TCA cycle. (B) Concentration of citrate catalyzed by CS (*n* = 6). (C) Concentration of α‐ketoglutarate (*n* = 6). (D) Concentration of fumarate (*n* = 6). (E) Concentration of malate (*n* = 6). (F) Concentration of 2‐hydroxyglutarate (*n* = 6). (G) Concentration of l‐lactate (*n* = 6). Mice in NaCr group were treated with NaCr (20 mg kg^−1^). Data were presented as the mean ± SD. ^*^ *p* < 0.05 versus DSS group; ^##^ *p* < 0.01 versus control group.

In the TCA cycle, each reaction is facilitated by a specific enzyme to ensure the smooth progression of the cycle, where the product of each step serves as the substrate for the subsequent reaction (Figure 3A). As shown in Table 2, the KCr level of enzymes involved in the TCA cycle was notably diminished in DSS‐induced UC mice. Hence, the aim of subsequent experiments was to identify the particular enzymes within the TCA cycle for which KCr is crucial for enabling the enzymes to carry out their catalytic function and drive the TCA cycle under UC conditions. Consequently, to determine the activities of CS, DLST, malate dehydrogenase (MDH), succinate dehydrogenase (SDHA), fumarate hydratase (FH), and lactate dehydrogenase (LDH), the level of their respective products was measured to evaluate the enzymatic function. The results indicated a significant reduction in the production of citrate catalyzed by CS in the DSS‐induced group, with a marked increase in citrate level observed following the induction of KCr by NaCr administration (Figure 3B). Conversely, the level of catalytic products of the other enzymes did not exhibit apparent changes (Figure 3C–G). The citrate metabolites of CS reduced in UC due to the down‐regulated KCr level of CS, increased KCr in CS enhances the production of citrate catalyzed by CS.

### 3.4 Elevated KCr Level Maintains Mitochondrial Homeostasis, Reduces Oxidative Stress and Inhibits Apoptosis

**FIGURE 4:**
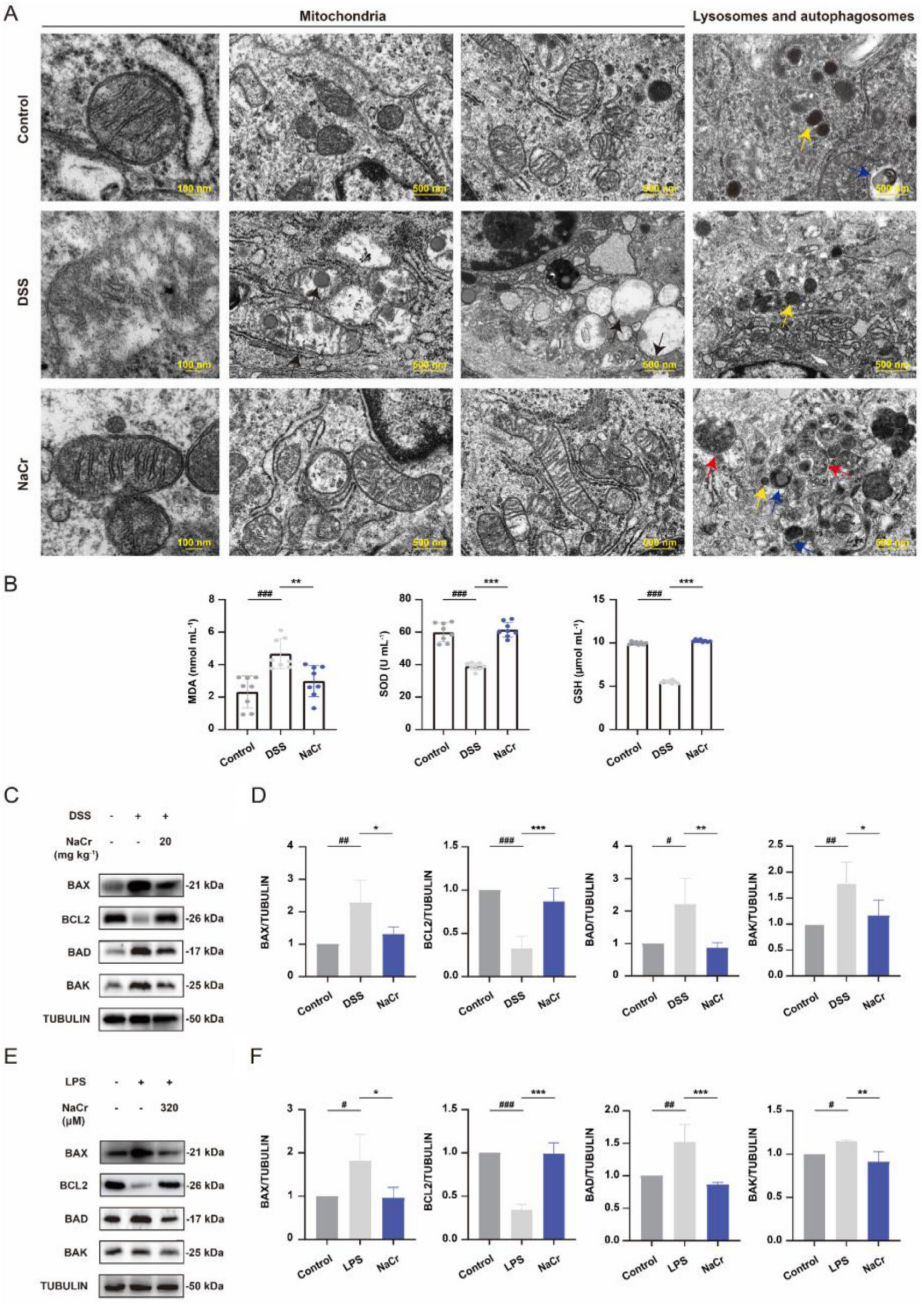
Elevated KCr level maintains mitochondrial homeostasis, reduces oxidative stress and inhibits apoptosis. (A) Representative TEM images of mitochondria, autophagosomes, and lysosomes in the colon tissue from control, DSS‐induced UC, and NaCr (20 mg kg^−^^1^)‐treated UC mice. Black arrows indicate mitochondrial swelling, edema and reduction or disappearance of cristae structures in the DSS‐induced group. Yellow arrows indicate lysosomes, blue arrows indicate autophagosomes, while red arrows indicate the process of mitophagy. (B) Oxidative stress markers (MDA content, SOD activity, and GSH content) in serum were tested by commercially available kits according to the manufacturer's instructions (*n* = 8). (C, D) Western blot and quantification of BAX, BCL2, BAD, and BAK with ImageJ (*n* = 4) in the colon tissue from control, DSS‐induced UC, and NaCr (20 mg kg^−^^1^)‐treated UC mice. (E, F) Western blot and quantification of BAX, BCL2, BAD, and BAK with ImageJ (*n* = 4) in NCM460 cells following the indicated treatment. Data were presented as the mean ± SD. ^*^ *p* < 0.05, ^**^ *p* < 0.01, ^***^ *p* < 0.001 versus DSS group; ^#^ *p* < 0.05, ^##^ *p* < 0.01, ^###^ *p* < 0.001 versus control group.

Previous studies highlighted the close association of KCr with mitochondria, next, TEM was employed to visualize the mitochondrial structure. As shown in Figure 4A, while mitochondria in the control group displayed typical mitochondrial morphology, characterized by a smooth outer membrane, an inner membrane connected to vesicular cristae and an electron‐dense, granular internal matrix, the mitochondria in the DSS‐induced group exhibited a perinuclear clustering, accompanied by the presence of damaged or absent cristae, fragmentation, and irregularities in the outer membrane, and mitochondrial damage was reduced after NaCr treatment. Damaged mitochondria was observed alongside mitophagosomes and autolysosomes [28]. The abundance of these structures was observed to increase after NaCr administration compared to DSS‐induced group (Figures 4A and S7). The equilibrium between mitochondrial function and oxidative stress is crucial for preserving normal cellular metabolism and preventing diseases. Oxidative stress is triggered when mitochondrial dysfunction leads to excessive ROS production or decreased antioxidant defenses [29]. Therefore, we assessed markers of oxidative stress. Compared with those in the control group, the level of MDA, SOD, and GSH in the serum of the DSS‐induced group exhibited notable alterations, while the SOD activity and GSH content were significantly increased and the content of MDA was significantly reduced in the NaCr group (Figure 4B). It is widely acknowledged that inflammation and oxidative stress have the potential to trigger apoptosis [30]. Thus, the expression level of apoptosis‐related proteins was assessed using Western blot and qPCR. In comparison to the control group, the expression of BAX, BAD, and BAK, pro‐apoptotic markers, were increased in colon tissue of the DSS‐induced UC group, whereas the increase in BAX, BAD, and BAK expression was attenuated in the NaCr group. Additionally, the expression of the anti‐apoptotic marker BCL2 was decreased in UC group, and elevated KCr significantly reversed the downregulation of BCL2 expression (Figure 4C–D). Next, we constructed a LPS‐induced UC model in NCM460 cell lines in vitro. Similarly, we observed a substantial increase in the down‐regulated expression of BCL2 following KCr enhancement (Figure 4E,F). These findings suggest that enhanced KCr preserves mitochondrial homeostasis, reduces oxidative stress and impedes apoptosis.

### 3.5 KCr Induces Mitophagy by Activating the PINK1/PARKIN Pathway

**FIGURE 5:**
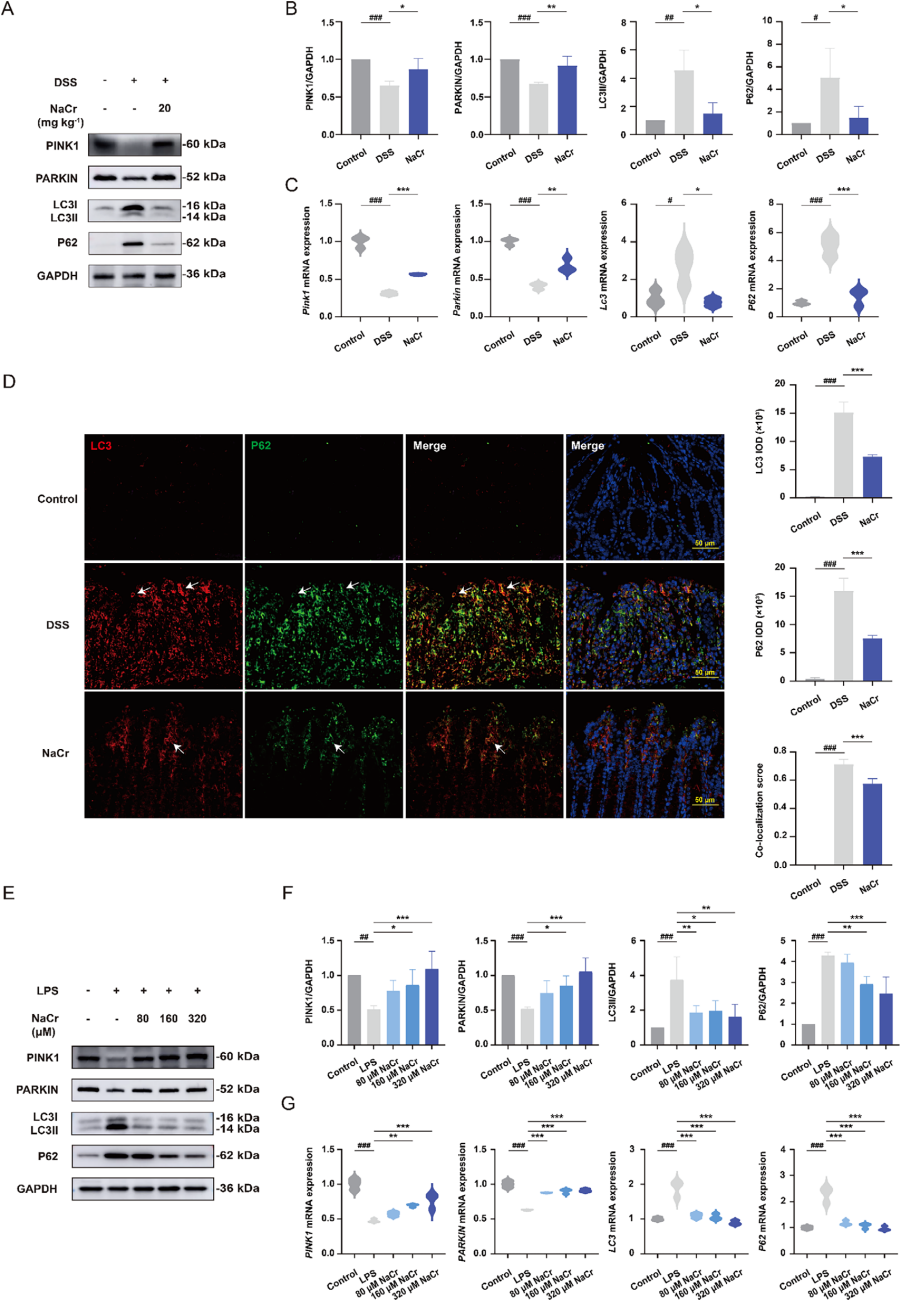
KCr induces mitophagy by activating the PINK1/PARKIN pathway. (A) Representative Western blot images of PINK1, PARKIN, LC3, and P62 in the colon tissue from control, DSS‐induced UC, and NaCr (20 mg kg^−1^)‐treated UC mice. (B) Quantification of PINK1, PARKIN, LC3, and P62 with ImageJ (*n* = 4). (C) The mRNA expression of *Pink1*, *Parkin*, *Lc3*, and *P62* in the colon tissue from control, DSS‐induced UC, and NaCr (20 mg kg^−1^)‐treated UC mice were determined by qPCR (*n* = 3). (D) Representative immunofluorescence images and quantification of LC3 and P62 in the colon tissue from control, DSS‐induced UC, and NaCr (20 mg kg^−1^)‐treated UC mice (*n* = 5). (E) Representative Western blot images in NCM460 cells following the indicated treatment. (F) Quantification with ImageJ (*n* = 4). (G) The mRNA expression of *PINK1*, *PARKIN*, *LC3*, and *P62* in NCM460 cells following the indicated treatment were determined by qPCR (*n* = 3). Data were presented as the mean ± SD. ^*^ *p* < 0.05, ^**^ *p* < 0.01, ^***^ *p* < 0.001 versus DSS group; ^#^ *p* < 0.05, ^##^ *p* < 0.01, ^###^ *p* < 0.001 versus control group.

Mitophagy is a critical process for maintaining mitochondrial homeostasis. We postulated that KCr could maintain the mitochondrial homeostasis and enhance mitochondrial function through mitophagy. The level of LC3 and P62, markers of autophagy, was evaluated by Western blot, qPCR and immunofluorescence co‐localization. Compared with control group, elevated level of both LC3II and P62 was observed in the DSS‐induced UC group, indicating a blockade in autophagic flux where the initiation of autophagic processes occurred but efficient substrate degradation was impeded, likely due to compromised autophagic clearance mechanism. Increased KCr level suppressed the elevation of P62 induced by DSS, indicating KCr triggered an unimpeded autophagic flow. However, LC3II expression was also reduced in the NaCr groups, the reason may be due to an excessive activation of autophagic flux (Figure 5A–D). We also observed that compared with control group, the expression level of LC3II and P62 was elevated in the LPS‐induced group. The upregulation of LC3II and P62 in LPS‐induced inflammation was attenuated by KCr, indicating the induction of autophagy by KCr (Figure 5E–G). Given that the majority of KCr was produced in mitochondria, while the mitochondria initiate mitophagy to preserve mitochondrial homeostasis. Additionally, the canonical PINK1/PARKIN‐mediated mitophagy pathway was examined. PINK1 and PARKIN are pivotal regulators involved in maintaining mitochondrial quality and are crucial for mitophagy. The level of PINK1 and PARKIN was down‐regulated in the colon of UC mice in comparison to the control group; conversely, elevated level of KCr resulted in the significant upregulation of PINK1 and PARKIN expression, indicating a positive effect of KCr in restoring mitophagy (Figure 5A–C). Also, the level of PINK1 and PARKIN was decreased in the LPS‐induced group compared to the control group, while the expression level of PINK1 and PARKIN was significantly elevated by KCr (Figure 5E–G). These observations suggest that mitophagy is induced by KCr through the activation of the PINK1/PARKIN pathway, both in DSS‐induced UC mice and in LPS‐induced inflammation.

### 3.6 KCr Restricts NLRP3 Inflammasome Activation by Inducing Mitophagy

**FIGURE 6:**
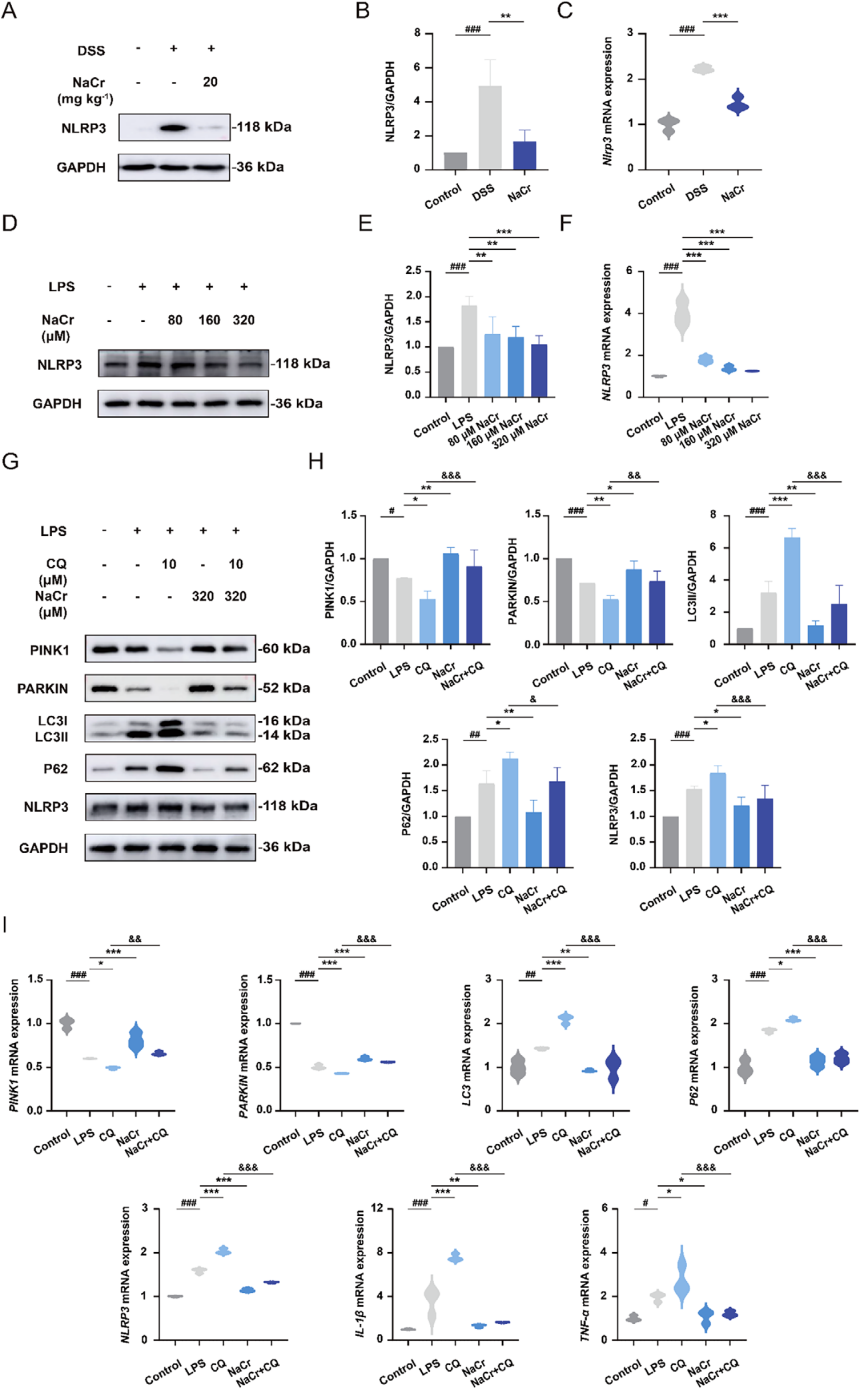
KCr restricts NLRP3 inflammasome activation by inducing mitophagy. (A) Representative Western blot images of NLRP3 in the colon tissue from control, DSS‐induced UC, and NaCr (20 mg kg^−1^)‐treated UC mice. (B) Quantification of NLRP3 with ImageJ (*n* = 4). (C) The mRNA expression of *Nlrp3* in the colon tissue from control, DSS‐induced UC, and NaCr (20 mg kg^−1^)‐treated UC mice were determined by qPCR (*n* = 3). (D) NLRP3 protein levels in NCM460 cells after indicated treatment. (E) Quantification of NLRP3 with ImageJ (*n* = 4). (F) The mRNA expression of *NLRP3* in NCM460 cells following the indicated treatment (*n* = 3). (G) Representative Western blot images of PINK1, PARKIN, LC3, P62, and NLRP3 in NCM460 cells following the indicated treatment. (H) Quantification of PINK1, PARKIN, LC3, P62, and NLRP3 with ImageJ (*n* = 4). (I) The mRNA expression of *PINK1*, *PARKIN*, *LC3*, *P62*, *NLRP3*, *IL‐1β*, and *TNF‐α* in NCM460 cells following the indicated treatmentwere determined by qPCR (*n* = 3). Data were presented as the mean ± SD. ^*^ *p* < 0.05, ^**^ *p* < 0.01, ^***^ *p* < 0.001 versus LPS group; ^#^ *p* < 0.05, ^##^ *p* < 0.01, ^###^ *p* < 0.001 versus control group, ^&^ *p* < 0.05, ^&&^ *p* < 0.01, ^&&&^ *p* < 0.001 versus CQ group.

The NLRP3 inflammasome is recognized as a key regulator in the pathogenesis of various inflammation‐related diseases, including UC [31]. Next, the modulatory effect of NaCr on NLRP3 was assessed through Western blot and qPCR. The results indicated that the expression of NLRP3 was increased in the DSS‐induced UC group compared to the control group, whereas enhanced KCr led to a decrease in NLRP3 expression (Figure 6A–C). Also, compared with control group, NLRP3 expression was elevated in LPS‐induced group, and the expression of NLRP3 was decreased by KCr (Figure 6D–F). Given the ability of NaCr to induce mitophagy, CQ was used to inhibit mitophagy [32]. Subsequently, the reduced of NLRP3 by KCr was reversed by CQ due to the mitophagy inhibition (Figure 6G–I). The results showed that inhibition of mitophagy by Mdivi‐1 was also effective in reversing the downregulation of NLRP3 by KCr (Figure S8). The NLRP3 inflammasome plays a crucial role in the inflammatory cascade, with its activation triggering a cascade of inflammatory responses that impact the generation and secretion of diverse inflammatory cytokines such as IL‐1β and TNF‐α [33]. The mRNA expression level of IL‐1β and TNF‐α in NCM460 cells was assessed by qPCR. Elevated level of KCr reduced inflammatory factor level, while treatment with CQ increased the release of inflammatory cytokines, indicating that increased KCr level induced mitophagy to decrease the production of inflammatory factors (Figure 6I). Therefore, these results suggest that the enhancement of KCr protects against NLRP3 inflammasome activation through the induction of mitophagy.

### 3.7 CS K375 Crotonylation Induces Mitophagy and Inhibits NLRP3 Inflammasome Activation

**FIGURE 7:**
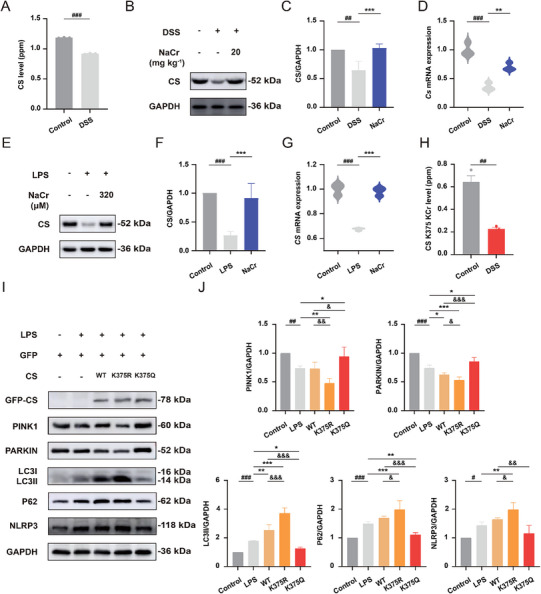
CS K375 crotonylation induces mitophagy and inhibits NLRP3 inflammasome activation. (A) The level of CS in DSS‐induced mice was detected by proteomics (*n* = 3). (B) Representative Western blot images of CS in the colon tissue from control, DSS‐induced UC, and NaCr (20 mg kg^−1^)‐treated UC mice. (C) Quantification of CS with ImageJ (*n* = 4). (D) The mRNA expression of *Cs* in the colon tissue from control, DSS‐induced UC, and NaCr (20 mg kg^−1^)‐treated UC mice were determined by qPCR (*n* = 3). (E) Representative Western blot images of CS in NCM460 cells following the indicated treatment. (F) Quantification of CS with ImageJ (*n* = 4). (G) The mRNA expression of *CS* in NCM460 cells following the indicated treatment (*n* = 3). (H) The level of CS K375 crotonylation in DSS‐induced mice was detected by PTM proteomics (*n* = 3). (I) NCM460 cells were treated with LPS (2 µg mL^−1^) after transfection with plasmids carrying CS with or without KCr site‐specific mutation from K to R or Q for 24 h. Representative Western blot images of PINK1, PARKIN, LC3 P62, and NLRP3 in NCM460 cells following the indicated treatment. (J) Quantification with ImageJ (*n* = 4). Data were presented as the mean ± SD. ^*^ *p* < 0.05, ^**^ *p* < 0.01, ^***^ *p* < 0.001 versus LPS group; ^#^ *p* < 0.05, ^##^ *p* < 0.01, ^###^ *p* < 0.001 versus control group, ^&^ *p* < 0.05, ^&&^ *p* < 0.01, ^&&&^ *p* < 0.001 versus WT group.

CS is a rate‐limiting enzyme in the TCA cycle. Biological processes that catalyze the condensation of oxaloacetic acid and acetyl‐CoA to produce citrate play important roles in the TCA cycle and are also involved in regulating mitochondrial homeostasis [34]. Proteomic analysis revealed a decreased level of CS in the colon tissue from DSS‐induced mice (Figure 7A). Subsequently, Western blot and qPCR analyses were employed to assess the expression level of CS. The results showed that the expression of CS was lower in DSS‐induced UC mice than in control mice, whereas enhancement of KCr led to an increase in CS expression (Figure 7B–D). Similarly, CS expression was reduced during LPS‐induced inflammation in comparison to the control group, while KCr elevated CS expression (Figure 7E–G). Additionally, a significant reduction in K375 crotonylation in CS was observed in DSS‐induced mice compared with the control group according to proteomic analysis (Figure 7H). To investigate the correlation between CS K375 crotonylation and mitophagy, site‐specific mutations were introduced in CS. Plasmids were utilized to transfect NCM460 cells with CS mutants in which K375 was substituted with arginine (K375R), a mutant that cannot undergo KCr at the K375 site, or glutamine (K375Q), a KCr‐mimicking mutant. Western blot was conducted to evaluate the impact of alterations in the K375 site in CS on mitophagy and NLRP3. The overexpression of wild‐type (WT) CS had a minimal effect on mitophagy in NCM460 cells. Conversely, mitophagy was significantly increased in NCM460 cells overexpressing the CS K375Q mutant compared to cells expressing the CS WT, while mitophagy was notably reduced in cells expressing the CS K375R mutant. However, compared with cells transfected with the CS WT mutant or GFP controls, cells transfected with the CS K375R mutant exhibited substantially impeded mitophagy following LPS stimulation (Figure 7I,J). By using NaCr treatment or rescuing the phenotype with the K375Q mutant in K375R‐transfected cells, we demonstrated that K375 is the crucial crotonylation site for the induction of mitophagy and inhibition of NLRP3 inflammasome activation (Figure S9). Therefore, it can be inferred that KCr induces mitophagy and inhibits NLRP3 inflammasome activation via the continuous activation of K375 site in CS.

## 4 Discussion

UC is a chronic, recurring, nonspecific inflammatory condition of the intestinal tract that primarily affects the mucosal layer of the colon. It typically starts in the rectum and may extend progressively to the distal part of the colon [35]. PTM, including ubiquitination, citrullination, N‐glycosylation and KCr, is a crucial mechanism for regulating protein function and plays a vital role in modulating the activity and physiological functions of diverse enzymes. The pathogenesis of UC involves abnormalities in PTMs of various proteins. For example, ubiquitin‐modifying enzymes impact intestinal barrier function and immune responses in UC through the regulation of ubiquitination [36]. Inhibitors of protein arginine deiminase are used to treat UC by inhibiting citrullination [37]. Furthermore, the modulation of N‐glycosylation by mesalazine enhances E‐calmodulin expression in colonic epithelial cells [38]. KCr is an important PTM that has been implicated in a wide range of diseases and biological processes, such as neuropsychiatric disorders, carcinogenesis, spermatogenesis, tissue injury and inflammation [39]. Nevertheless, the connection between KCr and UC remains unclear.

In this study, we initially assessed alterations in PTMs in the colon tissues of DSS‐induced UC model mice via Western blot analysis, and a notable change in the level of crotonylation was detected. Subsequently, WoLF PSORT software and KEGG enrichment analysis revealed that crotonylation occurs predominantly in the cytoplasm, nucleus and mitochondria, with the most enriched pathway (the TCA cycle) within the mitochondria. The TCA cycle is a critical component of energy metabolism in organisms, providing essential intermediates for various metabolic pathways. Maintenance of the TCA cycle and mitochondrial homeostasis is dependent on the expression of rate‐limiting enzymes within this pathway. PTM proteomic analysis revealed a decrease in the KCr level of key enzymes involved in the TCA cycle, such as CS in UC mice, suggesting a potential association between the crotonylation level of mitochondrial TCA cycle enzymes and UC. Next, NaCr was supplemented as a crotonylation inducer to increase the crotonylation level. Crotonylation induced by NaCr ameliorated symptoms such as weight loss, colon shortening and elevated secretion of inflammatory cytokines in a DSS‐induced UC mouse model. These findings suggested that increased level of crotonylation in colonic cells can impede the progression of UC‐related inflammation. Given that DSS‐induced UC mice exhibit altered crotonylation of enzymes primarily involved in the mitochondrial TCA cycle, we investigated the impact of crotonylation on catalytic product generation and maintenance of TCA cycle homeostasis by assessing the products of each enzymatic step. The results demonstrated that the catalytic activity of CS increased following the increase in crotonylation level. TEM observations revealed complete mitochondrial structure disruption in the UC model, and the effect was ameliorated by NaCr‐induced crotonylation, leading to improved mitochondrial morphology. Additionally, oxidative stress markers such as MDA, SOD, and GSH, as well as apoptotic indices related to mitochondrial function (BAX, BAD, BAK, and BCL2), were restored. Mitochondrial damage can increase oxidative stress, disrupt energy metabolism and cause cellular dysfunction and potentially cell death. To restore normal physiological function, cells engage in mitophagy to eliminate damaged and aged mitochondria and suppress inflammation. Subsequent Western blot and qPCR analyses demonstrated that increased crotonylation decreased abnormal autophagosome accumulation and activated the PINK1/PARKIN signaling pathway, suggesting its potential to induce mitophagy. Aberrant NLRP3 activation plays a crucial role in the pathogenesis of UC and mitophagy can impede the activation of the NLRP3 inflammasome [40]. Therefore, we further investigated whether crotonylation could suppress NLRP3 activation. Our findings demonstrated that an increase in crotonylation level led to a reduction in NLRP3 expression. Subsequently, using CQ and Mdivi‐1 to inhibit autophagy, we explored whether NaCr‐induced crotonylation could inhibit NLRP3 activation by promoting mitophagy. The results revealed that increased crotonylation decreased the expression of NLRP3 and reduced the production and release of inflammatory factors associated with NLRP3 activation. Building upon the investigation of the potential of pan‐crotonylation modifications to enhance UC and to gain a more detailed understanding of the precise role of these modifications in disease regulation at the molecular level, it is imperative to study crotonylation modifications on specific proteins and their specific K sites. In preliminary experiments, decreased crotonylation of CS was observed in DSS‐induced mice. Western blot analysis confirmed that CS expression was reduced in DSS‐induced UC mice and LPS‐induced inflammation compared with control group, and was elevated by enhanced crotonylation. To pinpoint the specific site of crotonylation, site‐specific mutations were constructed, focusing on the selected CS K375 site. Our results demonstrated that sustained activation of the CS K375 site could confer protection to NCM460 cells by inducing PINK1/PARKIN‐mediated mitophagy and inhibiting NLRP3 activation.

Crotonylation has emerged as a prominent subject of extensive research in recent years and is associated with the stability, activity or function of various proteins, including the tumor suppressor P53 and the autophagy protein ULK1 [41, 42]. This PTM has been linked to processes involved in inflammation, cancer and neurological diseases. The majority of studies on KCr have concentrated on its effects on histones (such as H1, H2A, H2B, H3, and H4), which play critical roles in regulating gene expression, chromatin structure and cellular processes [43]. However, investigations on non‐histone proteins have been limited, revealing effects on the function of proteins such as ACOX2, NPM1, DDX5, and ENO1 [44, 45]. A negative correlation was observed between crotonylation level and UC progression, suggesting that increased crotonylation may ameliorate UC by inducing mitophagy. The amino group of lysine can be diversely modified, including by acetylation, crotonylation, and other acylations, methylation, ubiquitylation, and ubiquitin‐like modifiers, this can result in competitive PTM crosstalk whereby different PTMs compete for modifying the same lysine residue [16]. Additionally, the K76 is frequently targeted for acetylation [46, 47, 48], while K366 is susceptible to both methylation and ubiquitination [49, 50]. Specifically, the crotonylation of K375 in CS was shown to play a crucial role in mitophagy. Crotonylation of K375 in CS prevented the decrease in CS expression in UC, thereby preserving the integrity of the mitochondrial TCA cycle and mitochondrial function. However, the specific regulatory relationship between the crotonylation of CS and CS expression remains unclear. Moreover, because NaCr is a general crotonylation inducer, the beneficial effects KCr on UC may involve not only elevated CS crotonylation but also other functional proteins, thus warranting further investigation. The precise link and underlying mechanism of interaction between crotonylation and mitophagy also need to be further explored.

In addition, NaCr showed no toxicity in body weight changes and DAI scores according to our research. The potential of NaCr to induce general crotonylation and ameliorate UC indicates that it may represent a novel therapeutic option for UC treatment. Furthermore, drugs targeting crotonylation modifications may present a promising avenue for clinical drug development in the management of UC.

## 5 Conclusions

In conclusion, this study presents the first comprehensive PTM‐proteomic analysis of protein KCr level in a DSS‐induced UC mouse model. Our findings offer novel insights into the therapeutic efficacy of increased crotonylation in alleviating symptoms of DSS‐induced UC. Mechanistically, increased crotonylation directly triggers the PINK1/PARKIN signaling pathway, attenuates the abnormal buildup of autophagosomes, suppresses NLRP3 activation and inhibits the release of associated inflammatory cytokines, contributing to the degradation of damaged mitochondria through mitophagy. Specifically, targeting KCr or CS may represent a promising therapeutic strategy for the management of UC.

## Author Contributions

Tongtong Liu designed research, performed research, analyzed data and wrote the paper. Binyan Lin wrote the paper. Jiayu Su, Ying Zhang, Xuan Wang, and Xiaochao Hu performed research. E‐Hu Liu and Shijia Liu designed research and contributed new analytic tools.

## Ethics Statement

All experiments involving animal studies were carried out according to the ethics policies and procedures approved by the Ethics Committee of Nanjing University of Chinese Medicine Affiliated Hospital (approval No. 2023NL‐KS222).

## Conflicts of Interest

The authors declare no conflicts of interest.

## Supporting information




**Supplementary File 1**: exp270088‐sup‐0001‐SuppMat.pdf.

## Data Availability

The data that support the findings of this study are available in the supplementary material of this article and from the corresponding author upon reasonable request.
