# Supplementary material for: Modulating Lysine Crotonylation in Ulcerative Colitis Maintains Mitochondrial Homeostasis: Modulating Crotonylation in Ulcerative Colitis
Source: Exploration (Beijing). 2025 Sep 19;5(6):20240129. doi: 10.1002/EXP.20240129 (PMC12752630; doi:10.1002/EXP.20240129)
Supplement: Supplementary file 1 — exp270088‐sup‐0001‐SuppMat.pdf. [file EXP2-5-20240129-s001.docx]

**Supporting Information**

**Modulating** **Lysine Crotonylation in Ulcerative Colitis Maintains Mitochondrial Homeostasis**

Tongtong Liu^a, b†^, Binyan Lin^c†^, Ying Zhang^a, b†^, Jiayu Su^a, b^, Xiaochao Hu^a, b^, Xuan Wang^a, b^, E-Hu Liu^c*^, Shijia Liu^a*^

*^a^ Affiliated Hosptial of Nanjing University of Chinese Medicine, Jiangsu Province Hospital of Chinese Medicine, 210029, Nanjing, China;*

*^b^ College of The First Clinical Medicine, Nanjing University of Chinese Medicine, 210023, Nanjing, China;*

*^c^ College of Pharmacy, Nanjing University of Chinese Medicine, 210023, Nanjing, China.*

*** Correspondence author at:** Affiliated Hosptial of Nanjing University of Chinese Medicine, Jiangsu Province Hospital of Chinese Medicine, Nanjing University of Chinese Medicine, 210029, Nanjing, China.

E-mail addresses: yfy0039@njucm.edu.cn (Shijia Liu); liuehu2011@163.com (E-Hu Liu).

† Equal contribution.

**Supplementary Figures**


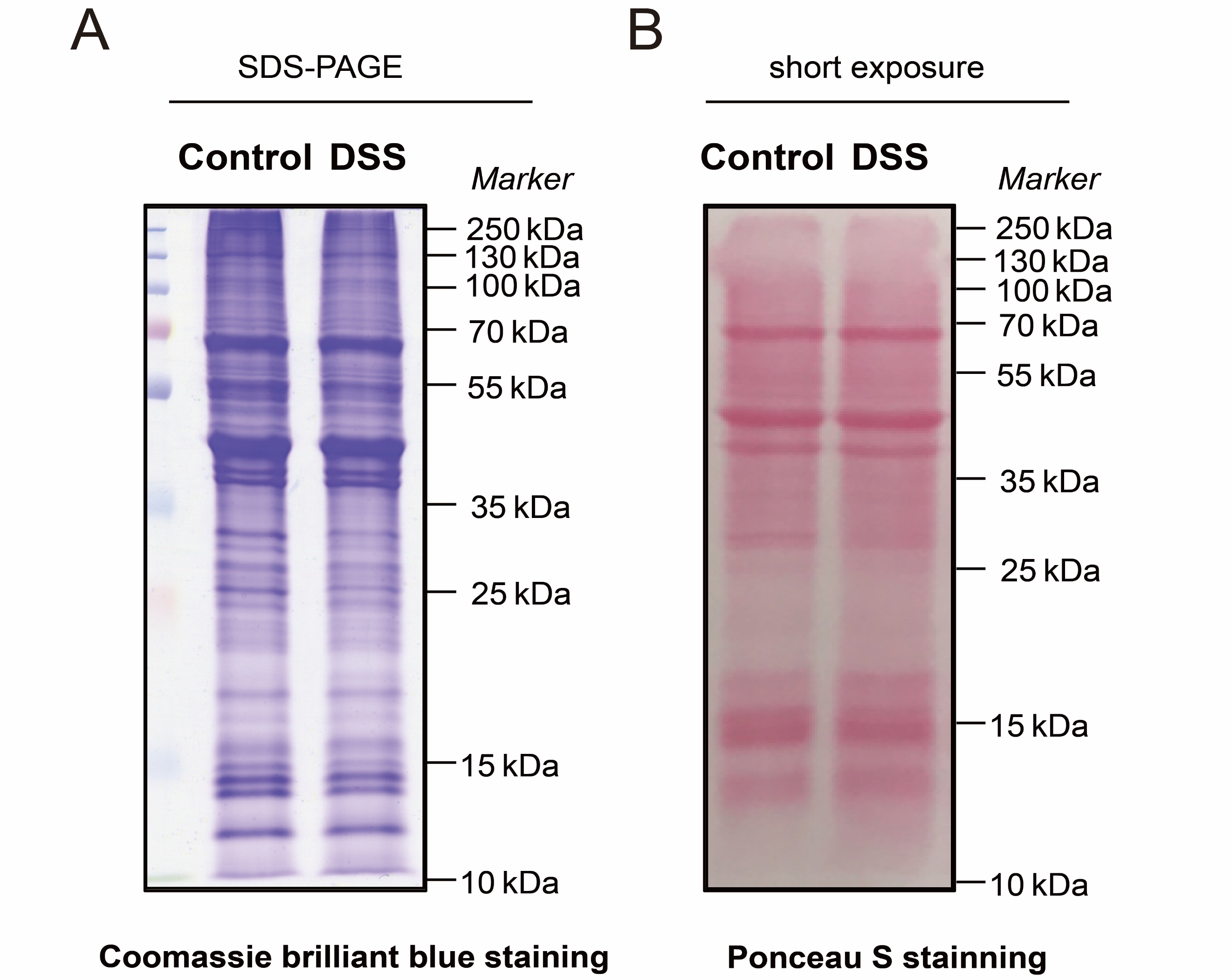


**Figure S1 (related to Figure 1). Representative western blot images of the internal control bands in the colon tissue between control and DSS-induced UC mice.** Twenty micrograms of sample were loaded onto each lane, and the lanes were verified to have equal protein loading per well using Coomassie brilliant blue staining (A) and Ponceau S staining (B).


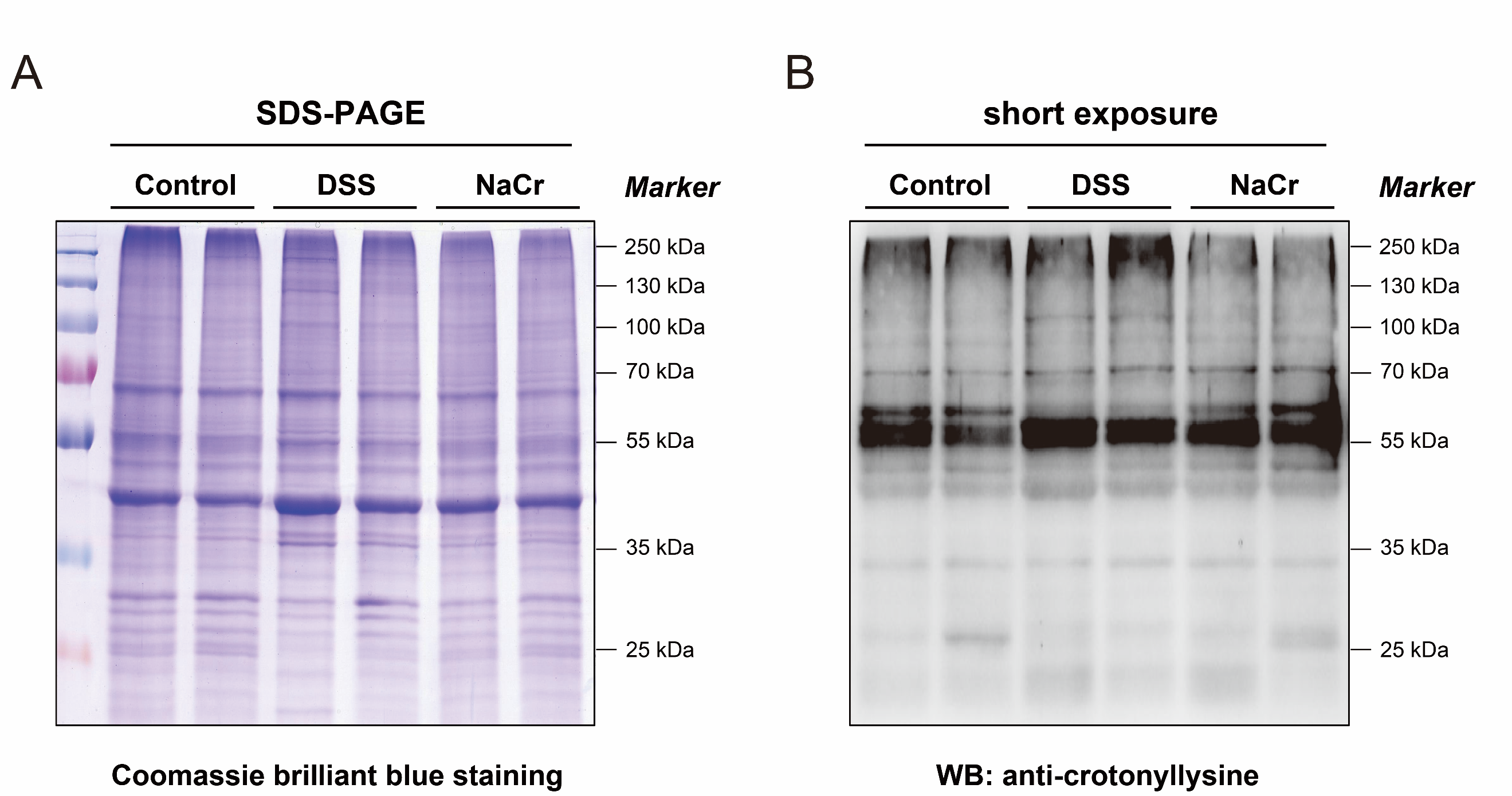


**Figure S2. NaCr promotes lysine crotonylation (KCr) level.** A. Coomassie brilliant blue staining. Mice in NaCr group were treated with NaCr (20 mg kg^-1^). B. Representative western blot image of crotonylation in the colon tissue of DSS-induced UC mice.


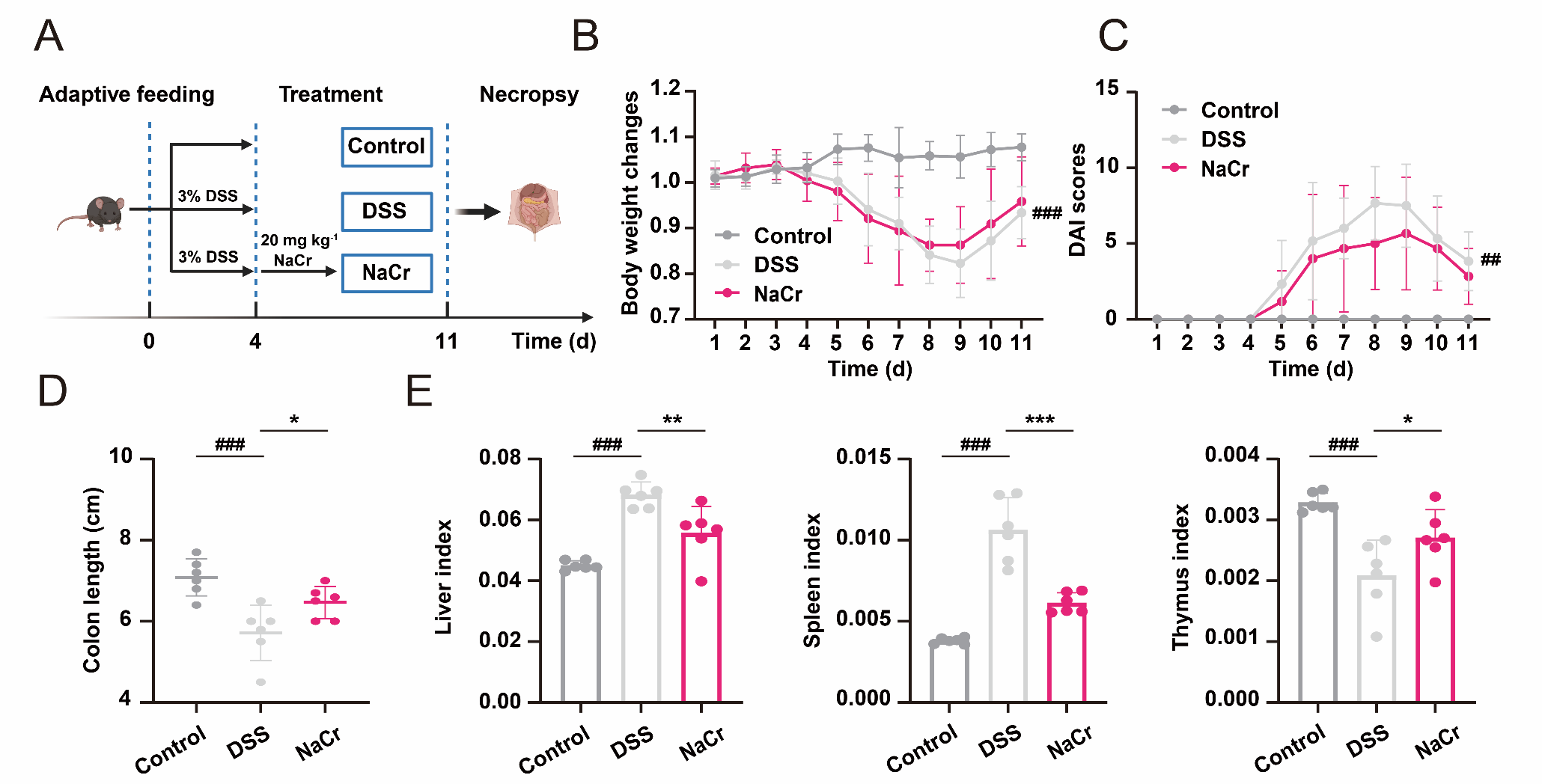


**Figure S3 (related to Figure 2). NaCr exhibits significant alleviation of DSS-induced UC after the establishment of UC disease model (n = 6).** A. Timeline of the animal experiment. Female C57BL/6 mice were first administrated 3% DSS for 4 days to induce UC model and treated with solvent control or NaCr (20 mg kg^-1^). B. Body weight changes. C. DAI scores. D. Length of colon tissues. E. Liver index, spleen index and thymus index. Data were presented as the mean ± SD. ^*^*P* < 0.05, ^**^*P* < 0.01, ^***^*P* < 0.001 *vs* DSS group; ^##^*P* < 0.01, ^###^*P* < 0.001 *vs* control group.


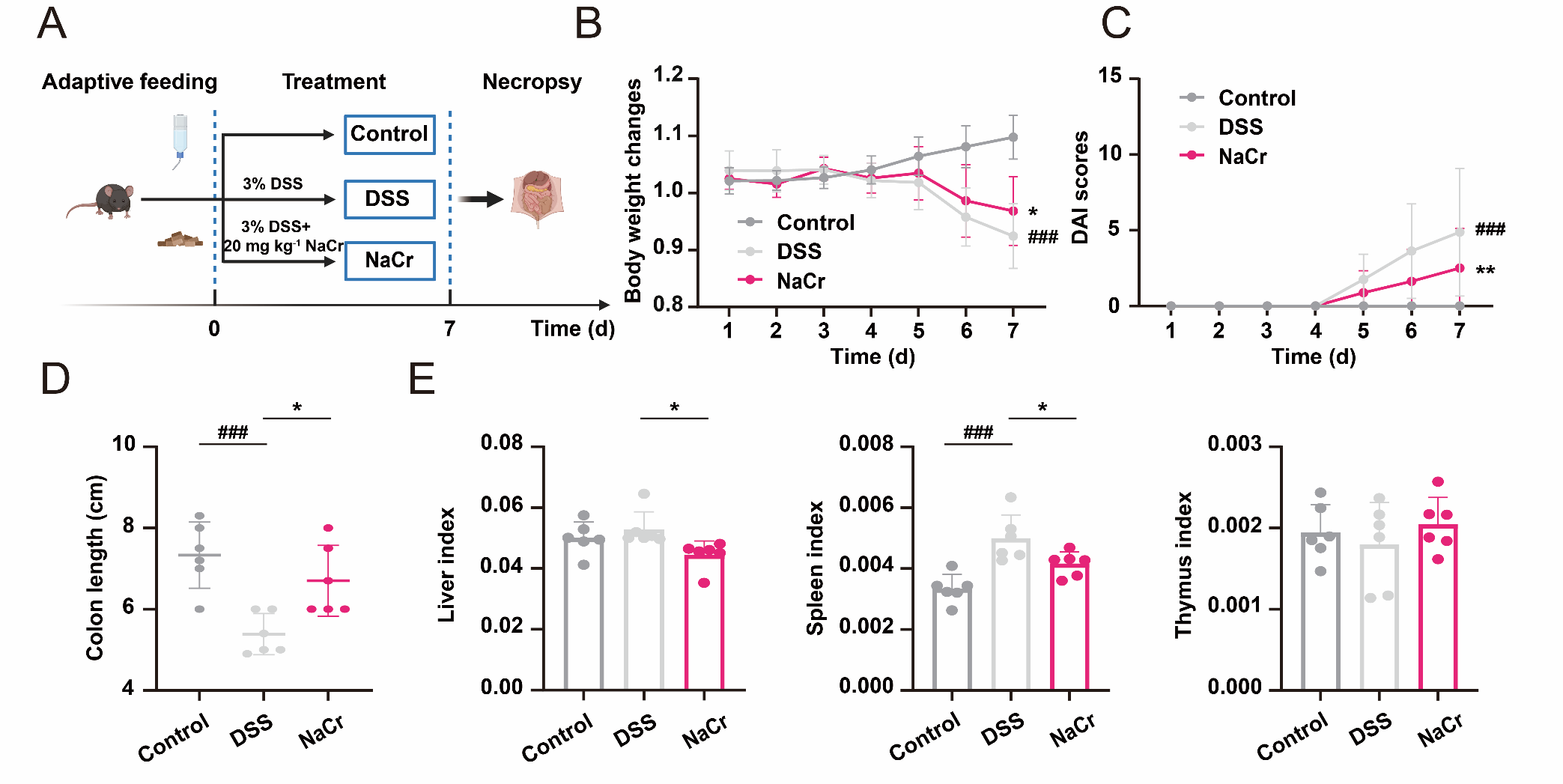


**Figure S4 (related to Figure 2). NaCr also impedes the inflammatory progression of UC model in male mice.** A. Timeline of the animal experiment. Male C57BL/6 mice in NaCr group were treated with NaCr (20 mg kg^-1^). B. Body weight changes (n = 8). C. DAI scores (n = 8). D. Length of colon tissues (n = 6). E. Liver index, spleen index and thymus index (n = 6). Data were presented as the mean ± SD. ^*^*P* < 0.05, ^**^*P* < 0.01 *vs* DSS group; ^###^*P* < 0.001 *vs* control group.


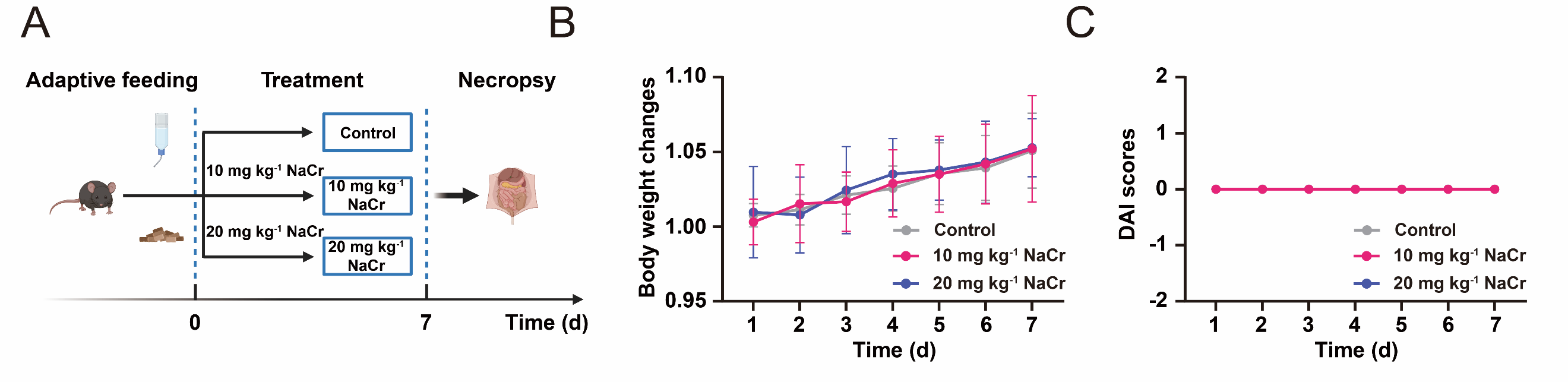


**Figure S5 (related to Figure 2). NaCr showed no toxicity at doses of 10 and 20 mg kg⁻¹.** A. Timeline of the animal experiment. Mice in NaCr group were treated with NaCr (10 or 20 mg kg^-1^). B. Body weight changes (n = 8). C. DAI scores (n = 8).


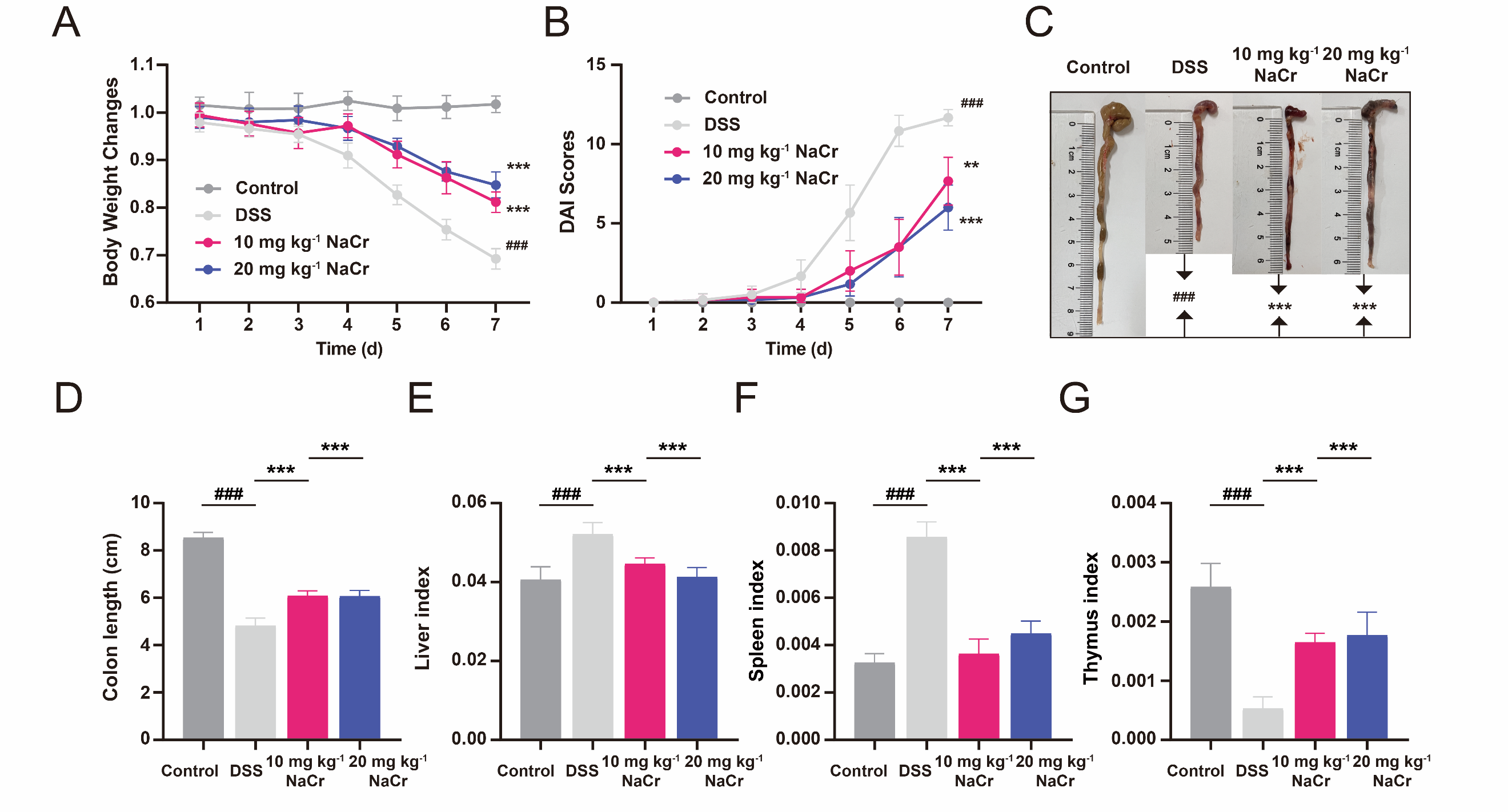


**Figure S6 (related to Figure 2). NaCr impedes the inflammatory progression of UC (n = 6).** A. Body weight changes. Mice in NaCr group were treated with NaCr (10 or 20 mg kg^-1^). B. DAI scores. C-D. Length of colon tissues. E. Liver index. F. Spleen index. G. Thymus index. Data were presented as the mean ± SD. ^**^*P* < 0.01, ^***^*P* < 0.001 *vs* DSS group; ^###^*P* < 0.001 *vs* control group.


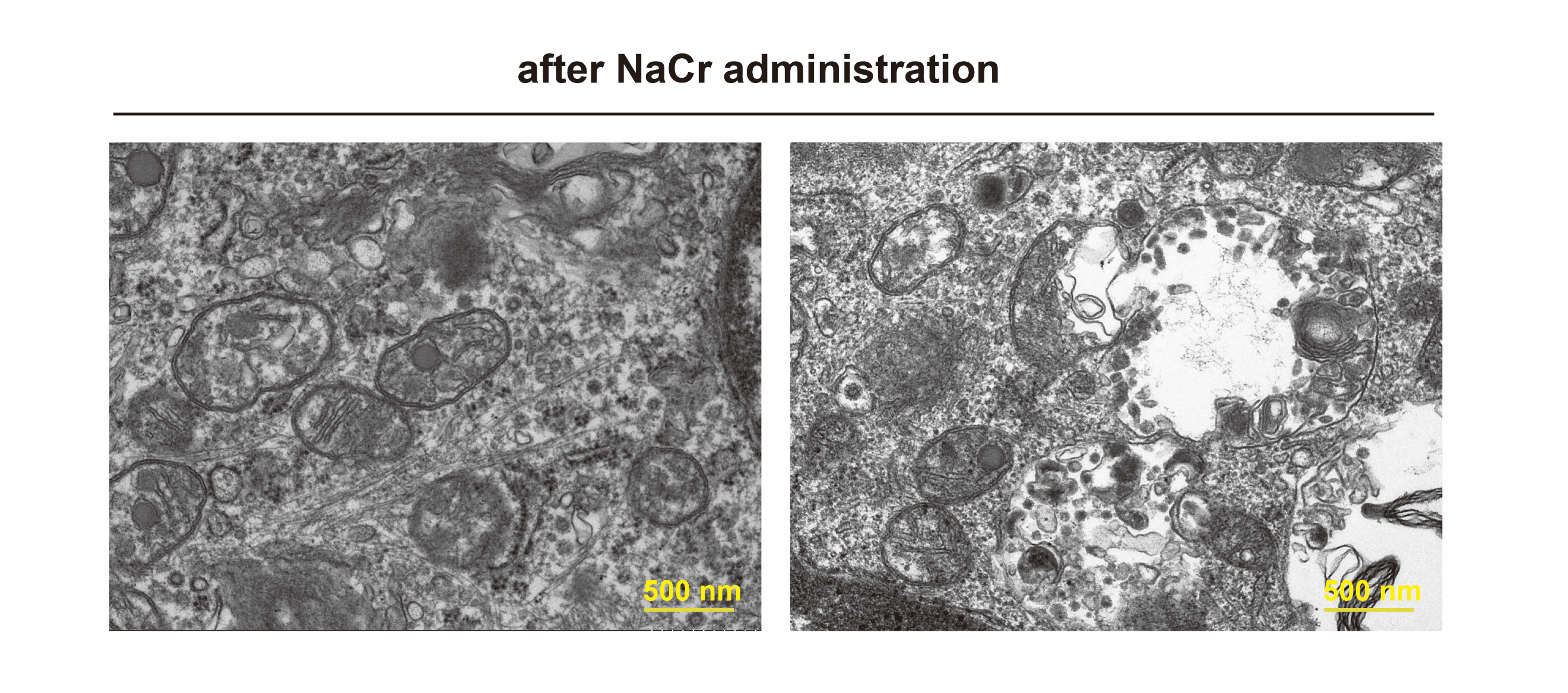


**Figure S7 (related to Figure 4). Elevated KCr level maintains mitochondrial homeostasis.** Representative TEM images of mitochondria, autophagosomes and lysosomes in the colon tissue from UC mice after 20 mg kg^-1^ NaCr administration.


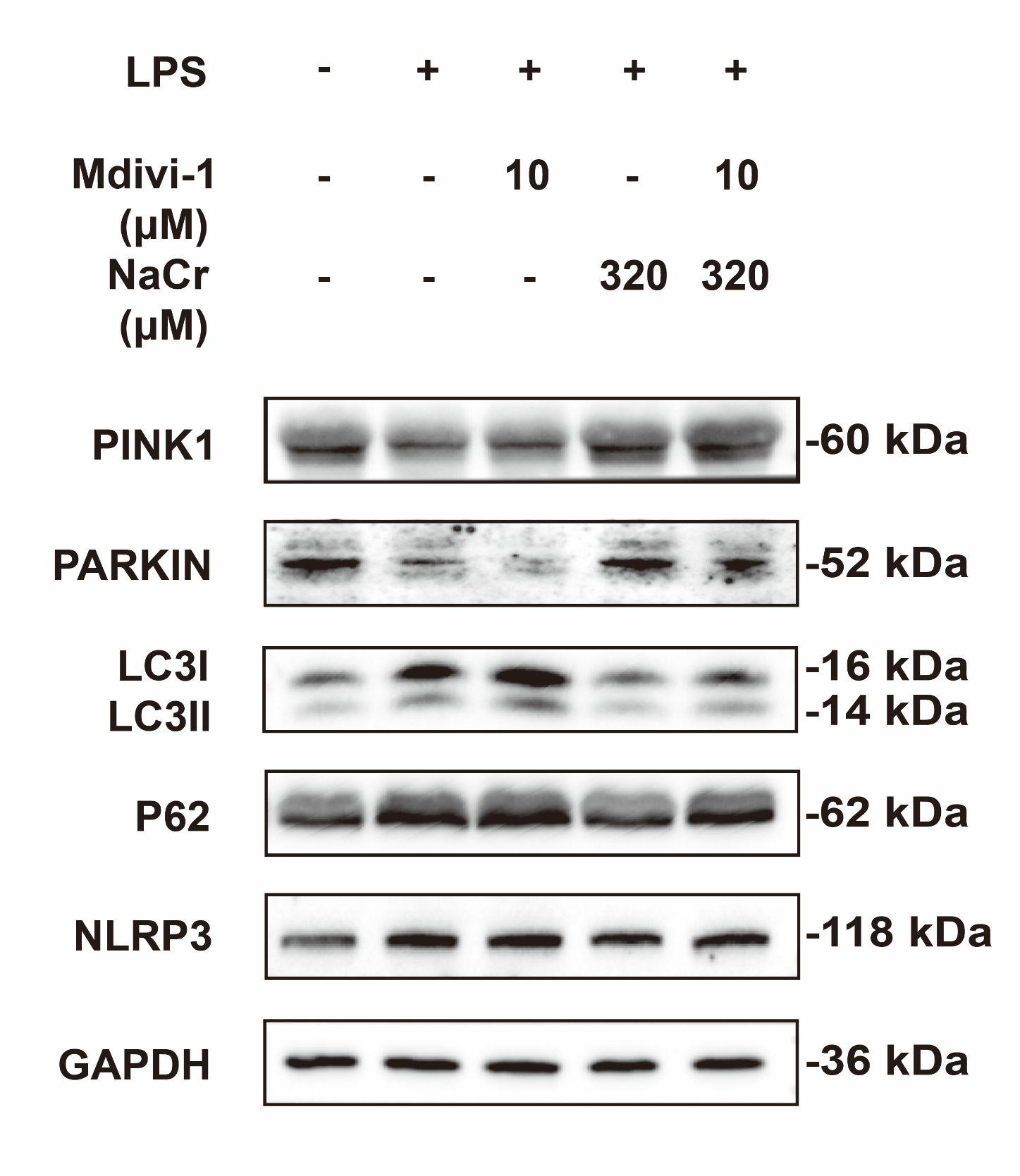


**Figure S8 (related to Figure 6). KCr restricts NLRP3 inflammasome activation by inducing mitophagy.** Representative western blot images of PINK1, PARKIN, LC3, P62 and NLRP3 in NCM460 cells following indicated treatment.


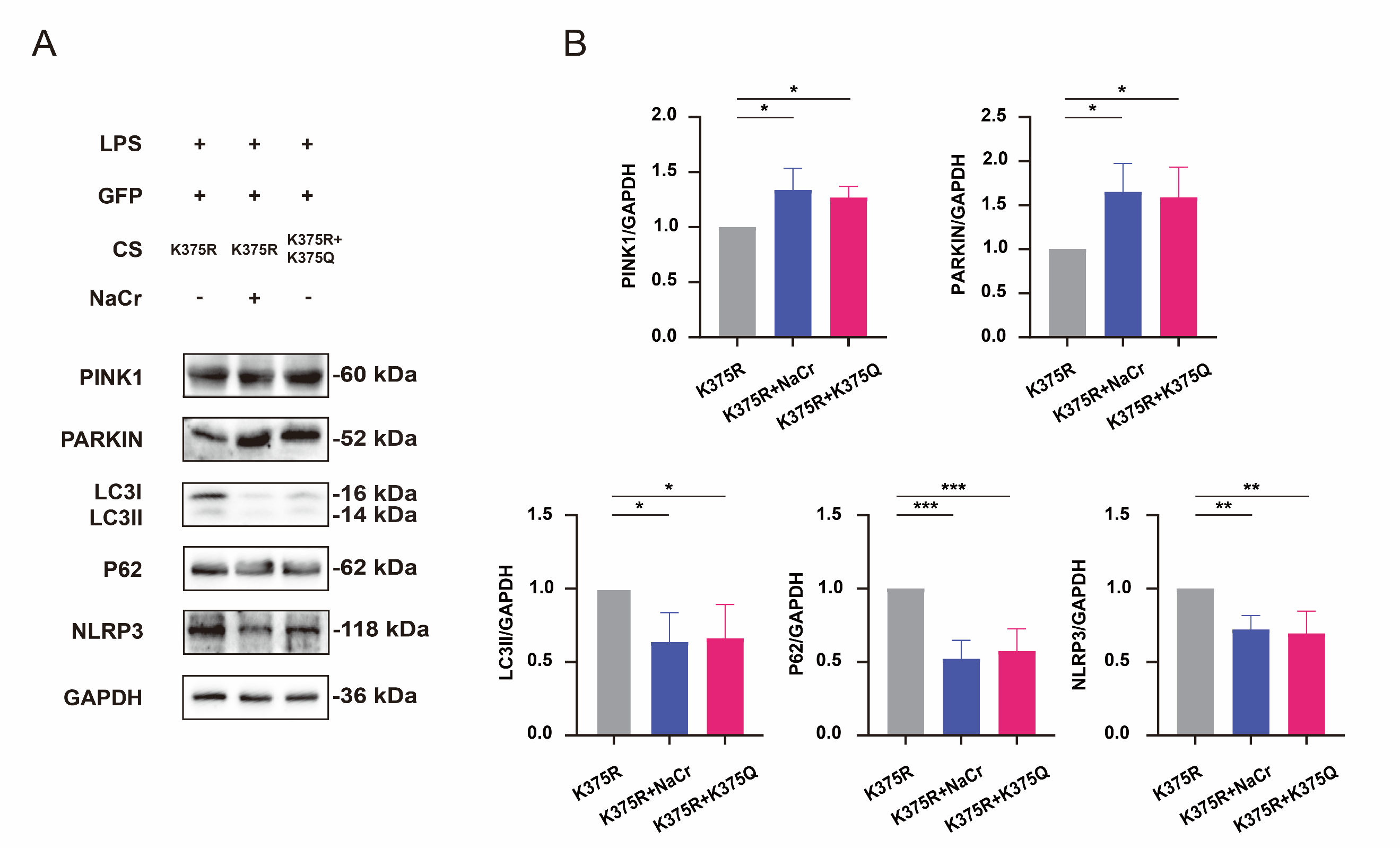


**Figure S9 (related to Figure 7). CS K375 crotonylation induces mitophagy and inhibits NLRP3 inflammasome activation.** A. NCM460 cells were treated with LPS (2 μg mL^-1^) after transfection with plasmids carrying CS with KCr site-specific mutation from K to R or Q for 24 h. Representative western blot images of PINK1, PARKIN, LC3 P62 and NLRP3 in NCM460 cells. B. Quantification with ImageJ (n = 4). Data were presented as the mean ± SD. ^*^*P* < 0.05, ^**^*P* < 0.01, ^***^*P* < 0.001 *vs* K375R group.
